# Application of Stem Cells in Oral Disease Therapy: Progresses and Perspectives

**DOI:** 10.3389/fphys.2017.00197

**Published:** 2017-04-03

**Authors:** Bo Yang, Yi Qiu, Niu Zhou, Hong Ouyang, Junjun Ding, Bin Cheng, Jianbo Sun

**Affiliations:** ^1^Guangdong Provincial Key Laboratory of Stomatology, Guanghua School of Stomatology, Sun Yat-Sen UniversityGuangzhou, China; ^2^State Key Laboratory of Ophthalmology, Zhongshan Ophthalmic Center, Sun Yat-Sen UniversityGuangzhou, China; ^3^Department of Spine Surgery, The Third Affiliated Hospital of Sun Yat-Sen University, Key Laboratory for Stem Cells and Tissue Engineering, Ministry of Education, Zhongshan School of Medicine, Sun Yat-Sen UniversityGuangzhou, China

**Keywords:** adult stem cells, iPS cells, oral and maxillofacial defect, stem cell therapy, clinical trial, precisely controlled differentiation

## Abstract

Stem cells are undifferentiated and pluripotent cells that can differentiate into specialized cells with a more specific function. Stem cell therapies become preferred methods for the treatment of multiple diseases. Oral and maxillofacial defect is one kind of the diseases that could be most possibly cured by stem cell therapies. Here we discussed oral diseases, oral adult stem cells, iPS cells, and the progresses/challenges/perspectives of application of stem cells for oral disease treatment.

## Introduction

**Figure 1:**
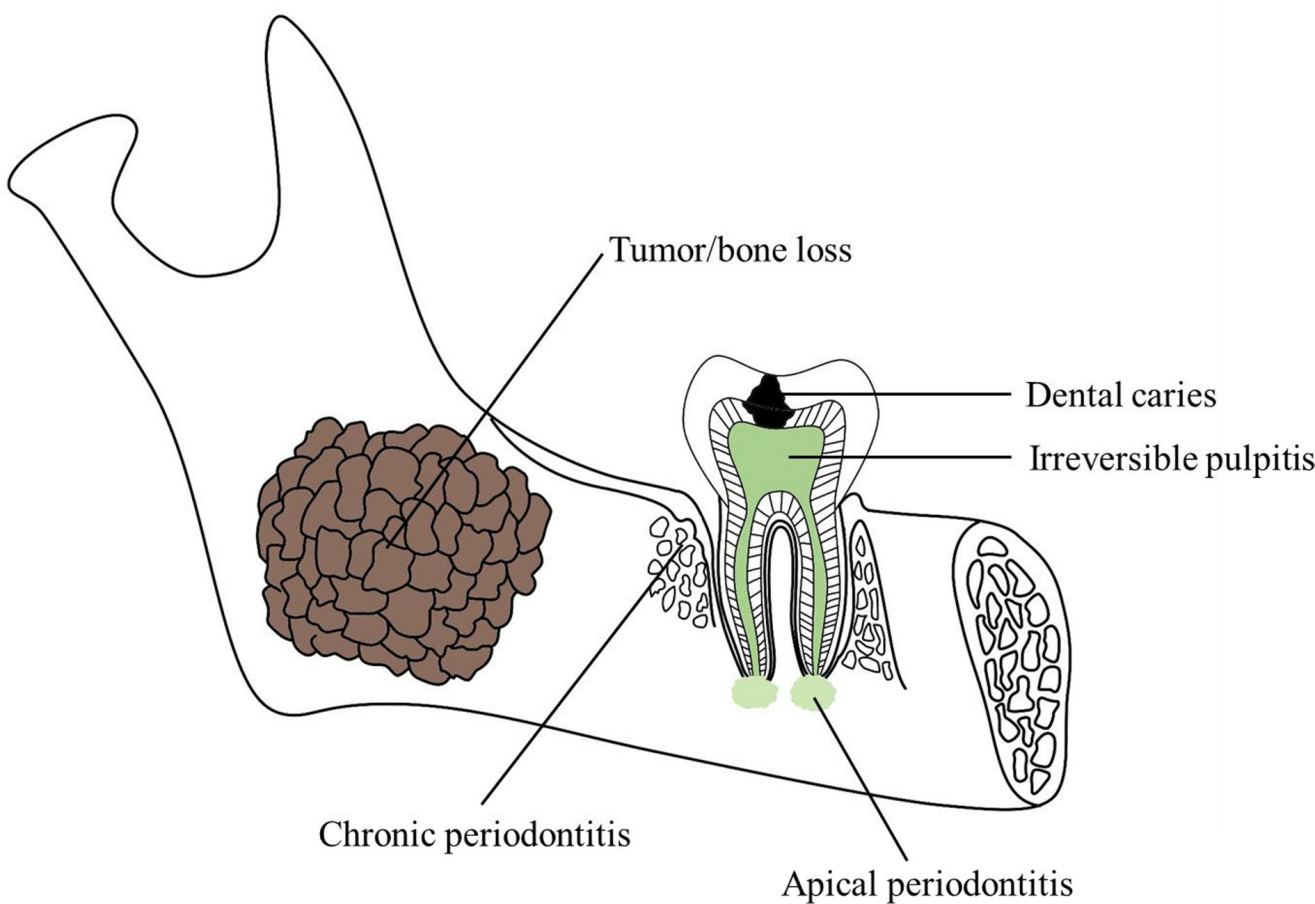
**Schematic of common oral and maxillofacial defects**.

Stem cells are undifferentiated and pluripotent cells that can produce more new stem cells (self-renewal) and differentiate into specialized cells with a more specific function such as skin cells, bone cells, and blood cells (Weissman, 2000). The major sources of stem cells include embryonic stem cells, adult stem cells, perinatal stem cells, and induced pluripotent stem cells (iPS cells). Stem cell research and clinical application has been dramatically advanced since the great invention of the iPS cell technology pioneered by Shinya Yamanaka in 2006 (Takahashi and Yamanaka, 2006). Stem cells have been reported for the therapy of multiple diseases such as leukemia, lymphoma, brain and spinal cord injury, heart failure, hearing loss, blindness and vision impairment, teeth missing, and other degenerative diseases. Oral and maxillofacial defect (Figure 1) is one kind of the diseases that could be most probably cured by stem cell therapy.

## Oral and maxillofacial defects

The oral and maxillofacial areas consist of many different types of tissues. It differs one from another and affects not only the functions of breathing, chewing, speech and smell, etc., but also esthetics and have much influence on the patients psychologically especially after accident injuries or tumor section (Mao and Prockop, 2012). The defects occurred in oral area can be divided into four main groups: dental hard tissue defect, pulpal disease, periodontal diseases, and maxillofacial defects (Table 1).

**Table 1:** **Oral and maxillofacial defects and their therapy**.

	**Dental hard tissue defects**	**Dental pulpal diseases**	**Periodontal diseases**	**Maxillofacial defects**
Features	Dental hard tissues turn dark, soft, or tissue loss caused by dental plaque, chemical erosion, trauma or abrasion	Dental pulpal inflammation or degradation caused by invasion of bacteria or stimuli of physical or chemical factors	Gingiva, periodontal ligament, alveolar bone, or cementum inflammation or loss caused by dental plaque, debris, or calculus	Maxillary or mandible bone, teeth in it, or soft tissue loss caused by tumor section, trauma, or congenital factors
Routine treatment	Amalgam or composite filling, occlusal veneer, or crown	Root canal therapy	Scaling and root planning; graft materials to compensate for the bone loss, barrier membranes for guided tissue regeneration, with bioactive molecules combined	Skin graft, flap reconstruction, or obturator rehabilitation
Stem cell therapy	No available reports	Dental stem cells, growth factors, and scaffolds have been respectively or conjunctively utilized to regenerate dental pulp and cementum-like, bone-like, or periodontal-like tissues could be generated in dental pulpal canals	Dental stem cells have been utilized in periodontal regeneration both in animal models and in clinical trials and proved to be safe	Mesenchymal and adipose-derived stem cells with scaffolds like calcium/phosphate-based bioactive ceramics and polymer based scaffolds, and bioactive factors like BMP, platelet-derived growth factors, and TGF-β, have been applied to defected sites
Perspectives	The assembly of amelogenin and other enamel matrix proteins, the proteolytic activity, and crystallization need to be in precise synergy with each other in order to produce the dental enamel	The clarification of the dental genetic modulating mechanisms, identification of good positional or cell lineage–specific markers of the dental pulp, the screening of appropriate scaffolds and the search for trophic factors maintaining are basic research objectives of dental bioengineering	Identification and isolation of the appropriate groups of stem cells and mimic a proper condition for their proliferation and differentiation are the first step. The delivering systems and scaffolds to support and facilitate their regenerative capacity are also of vital importance. Finally, in order for these cells to be used in humans, strict protocols according to the principles of Good Clinical Practice (GCP) and Good Manufacturing Practice (GMP) will be required	Suitable population of stem cells should be identified and harvested to fulfill the physiological role of the native tissue. Growth factors should be studied to support cellular differentiation and reproduction, and the role the microvasculature plays in tissue regeneration. Adverse events, such as infection, should be studied more deeply. Scaffolds should be investigated from the views of both clinicians and engineers
References	Uskokovic, 2015	Hargreaves et al., 2016; Mari-Beffa et al., 2017	Menicanin et al., 2015; Wang et al., 2015; Baba and Yamada, 2016; Chen et al., 2016; Panduwawala et al., 2016.	Krebsbach et al., 1997; Ueda et al., 2005; Rajan et al., 2014; Smith et al., 2015; Mele et al., 2016

### Dental defects

#### Dental hard tissue defects

Dental caries is one of the most common reasons to cause dental hard tissue defects. It is a multifactorial disease caused by the demineralization of tooth enamel surface by oral bacteria, thus weakening its structure (Soares et al., 2016). Non-carious lesions as a result of normal, abnormal, or pathological wear could cause abfraction, abrasion, and erosion or chemical degradation of dental tissues (Mjor, 2001). Dental traumatic injuries are caused by an external impact on a tooth and its surrounding tissues (Feliciano and de Franca Caldas, 2006). Up to now, direct filling is the main treatment method of dental hard tissues defects, in which either amalgam or composite resins would be used to restore the dental defects (Sequeira-Byron et al., 2015). However, there still exists problems like removing some healthy tissues to gain retention for amalgam (Ozcan et al., 2010) or shrinkage, easy to wear and aging, etc., for composite resins (Tantbirojn et al., 2011).

#### Pulpal diseases

Pulpitis refers to the inflammatory state of the dental pulp, clinically described as reversible or irreversible and histologically described as acute, chronic, or hyperplastic (Hargreaves et al., 2016). It often happens when there has been dental caries or trauma (Trowbridge, 1981). Root canal therapy has hitherto been applied to deal with pulpitis regularly. In this procedure, the inflammatory tissues are extracted, mechanical preparation of the root canals are carried out and then obturation would be done with filling materials to stop the bacteria from stepping into the root canal systems as well as the periodontal parts (Hargreaves et al., 2016). However, clearing up all the pulpal tissues leave the teeth insensitive, losing nutrients, and properties change of enamel and dentin.

#### Periodontal diseases

Periodontitis is the most common periodontal disease, which is a term referring to the inflammation of periodontium. Periapical periodontitis usually arises from inflammatory expansion from root canal systems. And more often, periodontitis origins from stimuli of oral bacteria. It would cause lesions of gingiva, attachment, alveolar bone and eventually the tooth loss (Caton et al., 2011). The methods to treat the periodontitis are subgingival scaling and root planning. However, the tissue loss can't be reversal, and these methods would only stabilize the situations.

### Maxillofacial defects

Maxillofacial tissues are important to individuals. People might have much trouble living confidently when suffering maxillofacial defects, usually caused by congenital anomalies, infections, trauma, and tumor section. Effective reconstruction or regeneration of the damaged part would be beneficial to patients both physiologically and psychologically (Mao and Prockop, 2012; Mertens et al., 2016). Flap transplants combined with dental implants or obturators are currently the main therapy methods to rehabilitate the defected tissues, however, the functions and esthetics still remain to be improved (Mertens et al., 2016; Okay et al., 2016; Wijbenga et al., 2016).

## Stem cells suitable for treatment of oral and maxillofacial defects

Stem cells are defined as the cells that can proliferate to achieve self-renewal and differentiate into multiple cell lineages (Weissman, 2000). There are four main types of stem cells: (1) embryonic stem (ES) cells from embryos; (2) adult stem cells from adult tissues; (3) perinatal stem cells existing in amniotic fluid; and (4) induced pluripotent stem cells (iPS cells) transformed from regular adult cells using genetic reprogramming. ES cells are the stem cells with the best pluripotency but ethical, legal, and medical considerations have hindered the development in clinical applications (Murray et al., 2007), especially when embryo has to be destructed to collect enough stem cells. Amniotic fluid stem cells need to be more studied to understand their potential. Therefore, we focus on the adult stem cells and iPS cells which could be used for the treatment of oral and maxillofacial defects.

### Oral-derived adult stem cells

Adult stem cells, also called somatic stem cells or postnatal stem cells (Tatullo et al., 2015), are harvested directly from adults. They are multipotent cells, only differentiating into limited types of cells or mesenchymal origin (Caplan, 1991). Dental tissues including bone marrow, dental pulp, dental follicle, periodontal ligament, apical papilla, and gingiva are good sources of adult stem cells. These cells commonly express specific molecular markers and can be isolated (Table 2). Recently, oral cavity have been paid more and more attention to gaining somatic stem cells as it's more flexible for the surgeons to harvest the tissues without undue trauma to the patients (Mao and Prockop, 2012).

**Table 2:** **Features and applications of oral-derived adult stem cells**.

	**Resources**	**Markers**	**Terminal cells**	**Applications (References)**
BMSCs	Bone marrow	STRO-1^+^ CD73^+^ CD90^+^ CD14^−^ CD34 ^−^ CD45^−^	Osteoblasts, Chondrocytes, Adipocytes, Cardiomyocytes, Myoblasts, and Neural cells	**Treatment of periodontitis in human; Bone Regeneration Using Bone Marrow Stromal Cells**; enhancement of new bone formation in immunocompromised mouse and rabbit models of distraction osteogenesis (DO); treatment of spinal cord injury in rat (Krebsbach et al., 1997; Menabde et al., 2009; Baba and Yamada, 2016; Ma et al., 2016; Okuda et al., 2017)
DPSCs	Dental pulp	CD105^+^ CD13^+^ CD73^+^ CD34^−^ CD45^−^	Odontoblast, Osteoblast, Chondrocyte, Cardiomyocytes, Neuron cells, Adipocyte, Corneal epithelial cell, Melanoma cell, Insulin secreting Beta cells	**Restore mandible bone defects in human**; bone regeneration in a rat critical-size calvarial defect model (d'Aquino et al., 2009a; Giuliani et al., 2013; Potdar and Jethmalani, 2015; Chamieh et al., 2016)
DFSCs	Dental follicle	CD44^+^ CD90^+^ CD150^+^ STRO-1^+^	Adipocytes, Osteocytes, Neural cells, Cementocytes, Periodonatal tissue	**Enhancement of bone regeneration on titanium implants surfaces in human**; cardiomyocyte differentiation and regeneration *in vitro* (Kemoun et al., 2007; Lucaciu et al., 2015; Sung et al., 2016; Lima et al., 2017)
Gingival	Gingival	CD146^+^ CD105^+^ CD34^−^	Osteoblasts, Adipocytes,	Periodontal regeneration in miniature-pigs; tendon regeneration in mouse model (Zhang et al., 2009; Moshaverinia et al., 2014; Fawzy El-Sayed et al., 2015; Fawzy El-Sayed and Dörfer, 2016)
PDLSCs	Periodontal ligament	STRO-1^+^ CD146^+^	Adipocytes, Cementoblasts, Osteoblasts, Neural crest-like cells	**Treatment of periodontal defects in human**; **tooth replacement**; cementum regeneration *in vitro*; tendon regeneration in mouse model (Feng et al., 2010; Gault et al., 2010; Moshaverinia et al., 2014; Chen et al., 2016; Cho et al., 2016)
SCAP	Apical papilla	STRO-1^+^ CD146^+^ CD34^−^ CD45^−^	Odontoblasts, Osteoblasts	Generation of cell-based three dimensional (3D) nerve tissue *in vitro* (Otsu et al., 2014; Kim B. C. et al., 2016)
SHEDs	Human exfoliated deciduous tooth	STRO-1^+^ CD44^+^ CD146^+^	Adipocytes, Odontoblasts, Neural cells, Osteoblasts	Generate a functional dental pulp when injected into full-length root canals *in vitro*; treatment of Alzheimer's disease (Rosa et al., 2013; Otsu et al., 2014)

**Figure 2:**
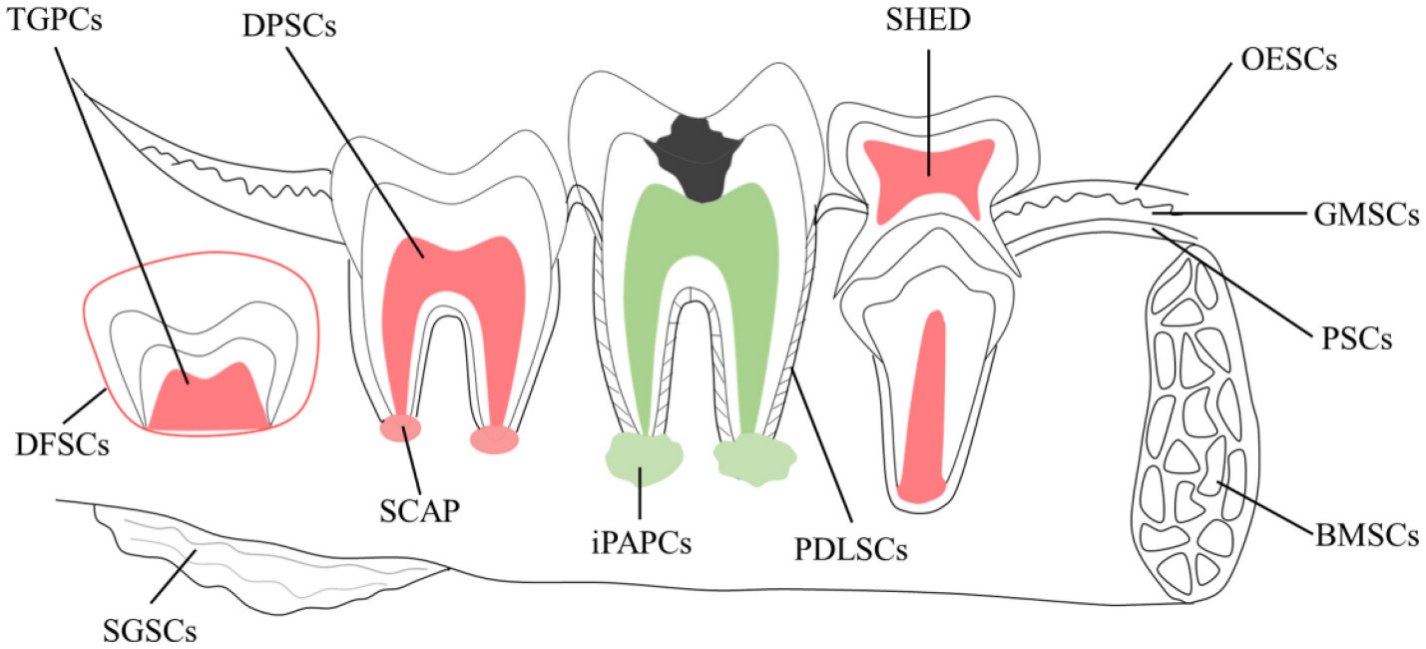
**Schematic of potential sources of adult stem cells in the oral environment**. Cell types include tooth germ progenitor cells (TGPCs); dental follicle stem cells (DFSCs); salivary gland stem cells (SGSCs); stem cells of the apical papilla (SCAP); dental pulp stem cells (DPSCs); inflamed periapical progenitor cells (iPAPCs); stem cells from human exfoliated deciduous teeth (SHED); periodontal ligament stem cells (PDLSCs); bone marrow stem cells (BMSCs); oral epithelial stem cells (OESCs); gingival-derived mesenchymal stem cells (GMSCs); and periosteal stem cells (PSCs).

There are different types of somatic stem cells in oral and maxillofacial compartments or “stem cell niches” (Figure 2). They are stem cells of the apical papilla (SCAP), inflammatory periapical progenitor cells (iPAPCs), dental follicle stem cells (DFSCs), dental pulp stem cells (DPSCs), periodontal ligament stem cells (PDLSCs), bone marrow stem cells (BMSCs), tooth germ progenitor cells (TGPCs), salivary gland stem cells (SGSCs), stem cells from human exfoliated deciduous teeth (SHED), oral epithelial stem cells (OESCs), gingival-derived mesenchymal stem cells (GMSCs), and periosteal derived stem cells (PSCs; Liao et al., 2011; Egusa et al., 2012). Most adult stem cells we could get in oral and maxillofacial areas are mesenchymal stem cells (MSCs). Pre-clinical studies of their utilization in treatment of several diseases have been extensively conducted in animal models (Table 2).

### Oral-derived induced pluripotent stem (iPS) cells

iPS cells are the pluripotent stem cells induced through reprogramming somatic cells genetically by introducing defined factors (Takahashi and Yamanaka, 2006). IPS technology is considered to be one of the most significant discovery and may make great contribution to the treatment of diseases. It brings hope to solve the ethical problems encountered by the application of human ES cells and limited material source for both ES cells and adult stem cells. Subsequently, a lot of studies have reported that defined transcription factors can directly induce one cell type converting to another without going through a pluripotent state, further promoting iPS cell technology (Zhou et al., 2008; Szabo et al., 2010; Vierbuchen et al., 2010; Huang et al., 2011; Kim E. Y. et al., 2016). During 10 year studies of iPS cells, extensive studies are focused on the multiple sides of iPS cells including method optimization, clinical application exploration, and the mechanisms of iPS cell reprogramming (Karagiannis and Eto, 2016). Most notably, iPS cell technology have made significant progresses in regenerative medicine that is to replace damaged organ part by establishing a normal functional cells, tissues or the whole organ (Mason and Dunnill, 2008).

**Figure 3:**
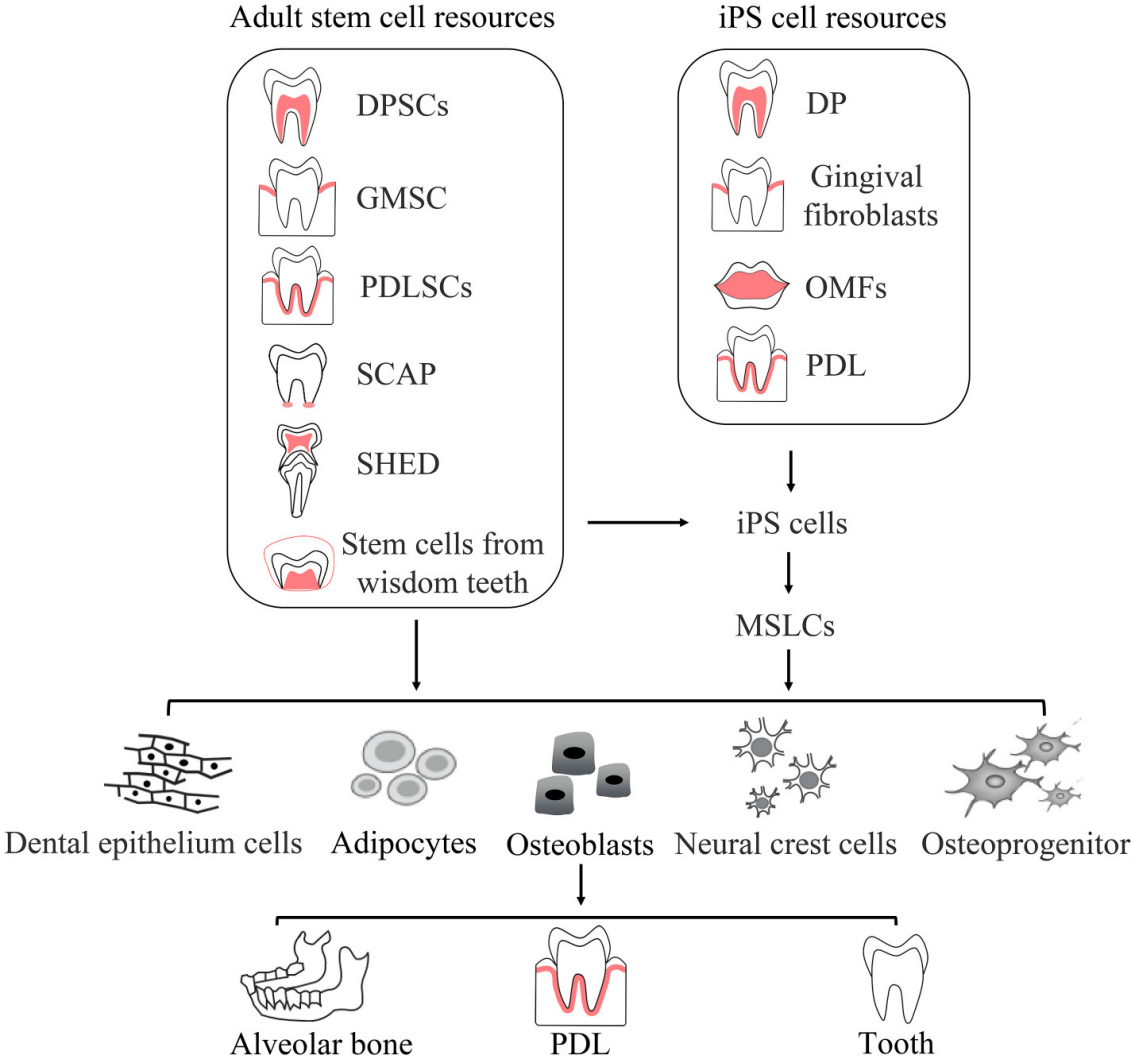
**Routine application of oral derived adult stem cells and iPS cells**. The figure shows dental resources of adult stem cells which, as well as fibroblast cells, are also resources of iPS cells. Adult stem cells directly differentiate into specialized cells or are induced into iPS cells. iPS cells induced from adult stem cells or fibroblast cells could be driven to differentiate into specialized cells. Specialized cells form final tissues and organs. DP, dental pulp; OMFs, Oral mucosa fibroblasts; MSLCs, Mesenchymal stem-like cells; PDL, periodontal ligament.

In the field of dentistry, for the regeneration of periodontal tissues, alveolar bone, and tooth are urgently needed. As iPS cell technology is considered to have great potentials, researchers are interested in the dental-tissue-derived iPS cells and the application of iPS cells in dental tissue regeneration. It was shown that three kinds of human dental stem cells: DPSCs, SHED, and SCAP, could be induced to generate iPS cells by introducing 3–4 factors with higher efficiency than human fibroblasts (Figure 3; Yan et al., 2010). Mesenchymal stromal cells derived from human third molars also can be reprogramed into iPS cells with high efficiency in a similar strategy (Oda et al., 2010). However, cells such as dental pulp and apical papilla are not convenient to isolate from patients for the donor tissues cannot regenerate. Human deciduous teeth might be discarded after exfoliation, so human deciduous tooth dental pulp cells (HDDPCs) are considered as better iPS cell source (Tamaoki et al., 2010). Using gingival fibroblasts as iPS cell source is another wise choice. Gingiva can be easily acquired from patients with no need of tooth or pulp extraction surgery and have relatively high reprogramming efficiency (Egusa et al., 2010; Wada et al., 2011; Arakaki et al., 2013).

## Applications of somatic stem cells in oral and maxillofacial repair and regeneration

Stem cell therapy has become a promising alternative in dentistry and maxillofacial rehabilitation since it could provide better physiological structure and functions (Rada et al., 2002; d'Aquino et al., 2009b; Caton et al., 2011; Tatullo et al., 2015). Regenerative dentistry aims to regenerate the damaged dental tissues and to regain the tooth morphology and functions (Figure 3 and Table 2). There are total 44 registered clinical trials correlated with oral stem cell or oral disease (Table 3). The stem cells used in the clinical trials include MSCs (*N* = 14, included five trials of BM-MSCs), PDLSCs (*N* = 4), OESCs (*N* = 12), DPSCs (*N* = 5), adipose derived stem cells (ADSCs; *N* = 6), SHED (*N* = 1), nasal stem cells (*N* = 1), and HSCs (*N* = 1). As outlined in Table 4, there are seven trials proposed to treat periodontal disease with autologous MSCs, ADSCs, PDLSCs, or allogeneic DPSCs. Three of them have reported results as shown in Table 4 and Supplementary Table 1. There are four clinical trials with reported results from total 14 trials for bone disease therapy with bone marrow stromal cells, nasal stem cells, allogeneic MSCs, and ADSCs. There are 11 trials for eye diseases with autologous OESC sheets but none has reported results yet. The other diseases with clinical trials include dental pulp diseases (*N* = 3, with autologous SHED or DPSCs), dental diseases correlated with tooth extraction (*N* = 2, treated with OESCs or DPSCs), graft vs. host diseases with oral complications (*N* = 2, treated by HSCs or MSCs), facial diseases (*N* = 2, with autologous ADSCs), and Xerostomia/Sjögren's Syndrome (*N* = 2, with autologous ADSCs or allogeneic MSCs). Among them, three trials have reported results. The clinical trials with reported results will be discussed below.

**Table 3:** **Stem cells used in the clinical trials correlated with oral disease and oral stem cell**.

**MSCs**	MSCs or BM-MSCs^*^	**PDLSCs or *BM-MSCs***	**OESCs or *HSC***	**OESCs or *nasal stem cells***	**DPSCs or *SHED***	**ADSCs**
**NCT02731586** (India, enrolling by invitation): Effect on Allogenic MSCs on Osseointegration of Dental Implants	NCT02751125^**^ (Norway, recruiting): Reconstruction of Jaw Bone Using MSCs	**NCT01357785** (China, completed): Periodontal Tissue Regeneration Using Autologous PDLSC (Chen et al., 2016)	**NCT02415218** (Japan, recruiting): Transplantation of Autologous OESC sheet for Limbal Stem-cell Deficiency	**UMIN000011290** (Japan, recruiting): Bone Regeneration Using Allogenic Secretomes	**NCT02523651** (China, recruiting): Periodontal Regeneration of Chronic Periodontal Disease Patients Receiving Stem Cells Injection Therapy	**UMIN000007698** (Japan, enrolling): An Exploratory Open Trial of Transplantation of ADSCs for Periodontal Regeneration
NCT00221130^**^ (Japan, completed): Clinical Trials of Regeneration for Periodontal Tissue using MSCs and Osteoblast Cells (Baba and Yamada, 2016)	**UMIN000011286** (Japan, completed): Bone Regeneration Using the Condition Media from Stem Cells	UMIN000005027^***^ (Japan, completed): Periodontal regeneration with autologous PDLSC sheets	**NCT03015779** (China, enrolling): Transplantation of Autologous OESC Sheet for Treating Limbal Stem Cell Deficiency Disease	**UMIN000012819** (Japan, recruiting): Cultivated Autologous OESC Sheet Transplantation	**UMIN000002050** (Japan, recruiting): Establishment and Analysis of Human Dental Pulp Stem Cells	**NCT02513238** (Denmark, enrolling): ADSCs for Radiation Induced Xerostomia (MESRIX)
UMIN000006720^**^ (Japan, published): Bone Augmentation with Tissue Engineered Bone (Ueda et al., 2005)	**NCT02755922** (Mexico, completed): Bone Regeneration With MSCs	NCT01082822^**^ (China, completed): PDLSC Implantation in the Treatment of Periodontitis	**NCT02739113** (Taiwan, onging): Cultivated Autologous OESCs Transplantation for the Treatment of Ocular Surface Diseases	**UMIN000012260, UMIN000012264** (Japan, no longer recruiting): Cultivated OESC Sheet Transplantation	**UMIN000016515** (Japan, recruiting): A Clinical Bone Regeneration Study with Dental Pulp Stem Cells	**NCT01309061** (South Korea, completed): The Effect of Human ADSCs in Romberg's Disease (Koh et al., 2012)
**NCT00953485** (China, recruiting): Allogeneic MSCs Transplantation for Primary Sjögren's Syndrome (pSS; Xu et al., 2012)	NCT02449005^**^ (Greece, active, not recruiting): Transplantation of Autologous BMSCs for the Regeneration of Infrabony Periodontal Defects	**NCT00172744** (Taiwan, completed): Effect of Cyclic Tensional Force on Osteogenic Differentiation of Human PDLSCs	**NCT00491959** (Taiwan, terminated): The Application of OESC Sheets Cultivated on Amino Membrane in Patients Suffering From Corneal Stem Cell Insufficiency or Symblepharon	**UMIN000005400, UMIN000006745** (Japan, no longer recruiting): Cultivated OESC Sheet Transplantation	**NCT02842515 (**France, completed**)**: Feasibility of the Preparation of an Advanced Therapy Medicinal Product for Dental Pulp Regeneration (Pulp'R)	**NCT02853942** (China„ not recruit yet): Autologous ADSCs Transplantation in the Treatment of Patients With Hemifacial Spasm
NCT01932164^**^ (Brazil, active, not recruiting): Use of MSCs for Alveolar Bone Tissue Engineering for Cleft Lip and Palate Patients	NCT00001391^**^(US completed): Bone Regeneration Using Bone Marrow Stromal Cells (Krebsbach et al., 1997)	NCT01389661^**^ (Spain, active, not recruiting): Treatment Of Maxillary Bone Cysts With Autologous BMSCs (MSV-H)	**NCT01489501** (France, withdrawn):Multicenter Study of CAOMECS Transplantation to Patients With Total Limbal Stem Cell Deficiency	**NCT02149732** (Korea, expanded access): Clinical Trial on the Effect of Autologous OESC Sheet Transplantation	**UMIN000009441** (Japan, active, no longer recruiting): Pulp regeneration with DPSCs	NCT02745379^**^ (Iran, phase I, recruiting): Effect of ADSCs in Maxillary Sinus Augmentation
**NCT02055625**(Sweden, recruiting): Treatment of Oral Mucosa in Patients With Graft-vs.-host Disease Following Injection of MSCs—Human Pilot Study	NCT00595595^**^ (US, completed): Development of a Model to Evaluate Regenerative Endodontic Techniques Using Extract Human Teeth	NCT02209311^**^(Russian, enrolling): Effectiveness and Safety of Method of Maxilla Alveolar Process Reconstruction Using Synthetic Tricalcium Phosphate and Autologous MMSCs	***NCT00023491** (US, completed): Potential of Transplanted Stem Cells to Mature Into Salivary Gland and Cheek Cells*	***NCT02900014** (France, recruiting): Validation of a Production Method of Stem Cell Isolated From the Nasal Cavity for an Innovative Cell Therapy of Cleft Palate*	***NCT01814436** (China, recruiting): Revitalization of Immature Permanent Teeth With Necrotic Pulps Using SHED Cells*	NCT02745366^**^ (Iran, recruiting): Buccal Fat Pad Derived Stem Cells with Cortical Tenting in Posterior Mandible Reconstruction

* *BM-MSCs: bone marrow MSCs*.

** *Stem cells combined with scaffolds*.

*** *https://www.researchgate.net/publication/266802767. The trials were identified through manually checking the cases obtained by searching “stem cell” combined with “oral,” “dental,” “pulp,” “periodontal,” “bone,” or “osteo” on the website of clinicaltrials.gov and umin.ac.jp*.

**Table 4:** **The diseases treated by stem cells in clinical trials correlated with oral disease and oral stem cells**.

**Target diseases**	**Stem cells**	Identifier (region, status)^*^	**Outcomes**
**Bone** diseases (craniofacial abnormality, mandibular fractures, bone atrophy, cleft palate, maxillary cyst and edentulous alveolar bone loss)	bone marrow stromal cells, nasal stem cells, allogeneic MSCs, ADSCs	NCT00001391^**^ (US, completed); **NCT02755922** (US, completed); NCT02751125^**^ (Norway, recruiting); **NCT02900014** (France, recruiting); NCT01389661^**^ (Spain, ongoing); **NCT02731586** (India, enrolling); NCT01932164^**^ (Brazil, active); **UMIN000016515** (Japan, recruiting); **UMIN000011286** (Japan, completed); **UMIN000011290** (Japan, recruiting); UMIN000006720^**^ (Japan, published); NCT02745379^**^ (Iran, recruiting); NCT02745366^**^ (Iran, recruiting); NCT02209311^**^ (Russia, enrolling);	4/14 of trials provided results. Prolonged bone formation by transplanted bone marrow stromal cells was observed in mouse models and consistent bone formation by human marrow stromal fibroblasts was achieved (NCT00001391) within vehicles containing hydroxyapatite/tricalcium phosphate ceramics (Krebsbach et al., 1997). MSCs from deciduous dental pulp associated with a collagen and hydroxyapatite biomaterial were used to reconstruct the alveolar bone defect in cleft lip and palate patients, and progressive alveolar bone union in all patients, final completion of the alveolar defect with an 89.5% mean bone height was detected 6 months postoperatively (NCT01932164). Application of stem cell derived growth factors for bone regeneration showed no side effects (UMIN000011286). The use of tissue engineered bone made of scaffolds and autogenous MSCs showed a mean increase in mineralized tissue height, indicating stable and predictable implant success (UMIN000006720; Ueda et al., 2005).
**Dental** pulp diseases	autologous SHED, DPSCs	**NCT01814436** (China, recruiting); **NCT02842515** (France, completed); **UMIN000009441** (Japan, no longer recruiting)	0/3 of trials provided results.
**Eye** diseases (Kerato conjunctival disease, corneal epithelial stem cell deficiency, limbal stem cell deficiency)	autologous OESC sheet	**UMIN000012819** (Japan, recruiting); **UMIN000012264**, **UMIN000012260**, **UMIN000006745**, **UMIN000005400** (Japan, No longer recruiting); **NCT02415218** (Japan, recruiting); **NCT03015779** (China, enrolling); **NCT02739113** (Taiwan, onging); **NCT00491959** (Taiwan, terminated); **NCT01489501** (France, withdrawn); **NCT02149732** (South Korea, expanded access);	0/11 of trials provided results.
**Facial** diseases (Injury of facial nerve, progressive hemifacial atrophy romberg's disease)	autologous ADSCs	**NCT02853942** (China, not recruiting yet); **NCT01309061** (South Korea, completed);	1/2 of trials provided results. Intramuscular autologous transplantation of ADSCs enhance the survival of fat grafted into the face in patient with progressive hemifacial atrophy (NCT01309061; Koh et al., 2012).
**GVHD** with oral complications	HSCs; MSCs	**NCT00023491** (US, completed); **NCT02055625** (Sweden, recruiting)	1/2 of trials provided results. Fifty five percent of aGVHD patients who failed front-line steroid treatment responded to MSC infusion (NCT02055625).
**Healthy**	autologous PDLSCs	**NCT00172744** (Taiwan, ongoing)	0/1 of trials provided results.
**Periodontal** diseases	autologous MSCs, ADSCs, PDLSCs; allogeneic DPSCs	NCT02449005^**^ (Greece, active); NCT00221130^**^ (Japan, completed); **NCT01357785** (China, completed); NCT01082822^**^ (China, completed); **NCT02523651** (China, recruiting); **UMIN000007698** (Japan, enrolling); **UMIN000005027** (Japan, completed);	3/7 of trials provided results. Implantation of autologous MSCs with a 3D woven-fabric composite scaffold and platelet-rich plasma showed no clinical safety problems but decreasing trend of mobility and significantly improved changes in clinical attachment level, pocket depth, and linear bone growth (NCT00221130; Baba and Yamada, 2016). Autologous application of PDLSCs in periodontitis patients no clinical safety problems but a significant increase in the alveolar bone height (decrease in the bone-defect depth) although no differences were detected between the Cell group and the Control group (NCT01357785; Chen et al., 2016). Autologous transplantation of PDLSCs showed no adverse reaction, reduction of pocket probing depth and clinical attachment gain, while greater linear bone gain at PDL cell sheets/β-TCP treated sites in the finished four cases (UMIN000005027)
**Teeth** extraction	OESCs, DPSCs	NCT00595595^**^ (US, completed); **UMIN000002050** (Japan, recruiting);	0/2 of trials provided results.
**Xerostomia**, Sjögren's Syndrome	autologous ADSCs; allogeneic MSCs;	**NCT02513238** (Denmark, enrolling); **NCT00953485** (China, recruiting);	1/2 of trials provided results. Intravenously infused allogeneic MSCs suppressed autoimmunity and restored salivary gland secretory function in both mouse models and Sjögren's Syndrome patients (NCT00953485; Xu et al., 2012).

* *From clinical trials. gov, and umin.ac.jp*.

** *Stem cells combined with scaffolds*.

### Somatic stem cells with scaffolds in oral and maxillofacial repair and regeneration

#### Scaffolds used in oral and maxillofacial repair and regeneration

Accurately designed scaffolds may improve the oral and maxillofacial regeneration (Lee et al., 2010; Mitsiadis et al., 2012; Hayashi et al., 2015). Scaffolds in oral and maxillofacial regeneration are three-dimensional (3D) biomaterials mimicking extracellular matrix facilitating cell-scaffold interactions, cell survival, proliferation, and differentiation. Thus, the scaffolds are mainly made of degradable and low toxic materials (Horst et al., 2012). There are four main types of scaffolds including natural polymers, synthetic polymers, calcium phosphate-based ceramic scaffolds, and composite scaffolds. More details of chemical structure, features and applications of scaffolds could be found in the literature (Mele et al., 2016). Scaffold materials are often applied together with stem cells and bioactive factors such as bone morphogenetic proteins (BMPs; Luu et al., 2007), vascular endothelial growth factor (VEGF; Schipani et al., 2009), platelet-derived growth factor (PDGF; Fiedler et al., 2004; Phipps et al., 2012), and SDF-1 (Kitaori et al., 2009). More details about growth factors used for craniofacial and bone regeneration could be found in the recent review (Mele et al., 2016).

The stem cells responded differently to various types of scaffolds (Motamedian et al., 2016). Eluted zinc released from zinc-modified titanium which is frequently applied in dental and maxillofacial implantation could stimulate osteoblast differentiation of DPSCs (Yusa et al., 2016a,b). Mangano et al. found that laser sintered titanium surface enhanced DPSCs to quickly differentiate into osteoblasts and endotheliocytes and then produce bone tissues along the implant surfaces. Eventually, a complete osteointegration was obtained (Mangano et al., 2010). After hooking into the biocoral scaffolds, DPSCs moved into the cavities and differentiated into osteoblasts, forming an engineer biocomplexs (Mangano et al., 2011). Giuliani et al. used the Micro-CT as an effective tool to observe the proliferation rate of different cells on the PLGA scaffolds (Giuliani et al., 2014). The porous PLGA microscaffolds have been proved to enhance the adhesion of DPSCs, meanwhile maintaining the viability, stemness, and plasticity of the cultured dental pulp mesenchymal stem cells (Bhuptani and Patravale, 2016). And the scaffold morphology was also confirmed to influence the long-term kinetics of bone regeneration (Giuliani et al., 2016).

#### Somatic stem cells with scaffolds in dental repair and regeneration

Stem cells and scaffolds could be transferred to dental canal systems to help regenerate vital pulp and continue root formation (Chrepa et al., 2015). Yadlapati et al. showed that VEGF-loaded fiber was biocompatible and might be a promising scaffold for additional optimization and use in endodontic regenerative procedures (Yadlapati et al., 2017). Theocharidou et al. proved low-level laser irradiation treatment to be beneficial for odontogenic differentiation and biomineralization of DPSCs inside the bioceramic scaffolds, making this therapeutic modality promising for targeted dentin engineering (Theocharidou et al., 2016). DPSCs and treated dentine matrix scaffolds were found to associate with significantly more bone formation when used to repair uninfected furcation perforations in the premolar teeth of dogs (Bakhtiar et al., 2016).

There are 12 cases using scaffolds for stem cell therapy among total 44 clinical trials correlated with oral disease and oral stem cells (Tables 3, 4). Eight of them are for the treatment of bone diseases including craniofacial abnormality, mandibular fractures, bone atrophy, cleft palate, maxillary cyst, and edentulous alveolar bone loss (five ongoing trials plus three trials with reported results) and three for periodontal diseases (NCT02449005, NCT00221130, NCT01082822), plus one for *in vitro* regeneration of dental pulp-like tissue with various scaffold and oral mucosa obtained during surgical tooth extractions (NCT00595595). Bio-Oss scaffolds were transplanted together with PDLS cell sheets for the chronic periodontitis therapy in a completed clinical trial (NCT01082822). Commercially available collagen scaffolds (collagen fleece) are used to hold autologous BM-MSCs enriched with autologous fibrin glue in clean room facilities for regeneration of periodontal tissues in periodontal infrabony defects in an ongoing clinical trial (NCT02449005). For adult periodontitis patients, the surgical implantation of autologous MSCs with a 3D woven-fabric composite scaffold and platelet-rich plasma showed no clinical safety problems but decreasing trend of mobility and significantly improved changes in clinical attachment level, pocket depth, and linear bone growth (NCT00221130; Baba and Yamada, 2016).

#### Somatic stem cells with scaffolds in maxillofacial repair and regeneration

Stem cells combined with scaffold could regenerate bones effectively (Kitamura et al., 2011; Windisch et al., 2012). And plastic compression of collagen scaffolds seeded with DPSCs was shown to enhance the osteogenic differentiation of DPSCs as it increased the collagen fibrillary density in a rat critical-size calvarial defect model (Chamieh et al., 2016). In a clinical trial (NCT00001391) to examine the potential of cultured human bone marrow stromal cells which will ultimately be used to graft into craniofacial osseous defects, prolonged bone formation by transplanted bone marrow stromal cells was observed in mouse models and consistent bone formation by human marrow stromal fibroblasts was achieved within vehicles containing hydroxyapatite/tricalcium phosphate ceramics (HA/TCP) in the form of blocks, powder, and HA/TCP powder-type I bovine fibrillar collagen strips (Krebsbach et al., 1997). Another clinical trial (UMIN000006720) evaluated the use of tissue engineered bone made of scaffolds and autogenous MSCs for maxillary sinus floor augmentation or onlay plasty with simultaneous implant placement in six patients with 3–5 mm alveolar crestal bone height. Results showed a mean increase in mineralized tissue height of 7.3 ± 4.6 mm postsurgically, indicating that injectable tissue-engineered bone provided stable and predictable implant success (Ueda et al., 2005). A clinical study demonstrated that a DPSC/collagen sponge biocomplex could completely restore human mandible bone defects and indicated that this cell population could be used for the repair and/or regeneration of tissues and organs (d'Aquino et al., 2009a). It is reported that 3 years after transplants in human mandibles, histological, and in-line holo-tomography revealed that stem cells regenerated a compact rather than a spongy bone. It created steadier mandibles, which might well-increase implant stability and resistance to mechanical, physical, chemical, and pharmacological agents (Giuliani et al., 2013). MSCs from deciduous dental pulp associated with a collagen and hydroxyapatite biomaterial (Geistlich Bio-Oss®) were used to reconstruct the alveolar bone defect in cleft lip and palate patients, and the results showed progressive alveolar bone union in all patients, final completion of the alveolar defect with an 89.5% mean bone height 6 months postoperatively (NCT01932164).

The other five clinical trials using scaffolds for stem cells are ongoing without reported results. An ongoing clinical trial (NCT01389661) uses a bioengineered product composed of mesenchymal stem cells and a patented cross-linked matrix of autologous plasma for bone maxillary cysts refilling. Platelet rich fibrin (PRF) and bone allograft (FDBA; SureOss, Hansbiomed, Korea) are used to load the buccal fat pad derived stem cells for the treatment of alveolar bone loss in maxillary sinus augmentation (NCT02745379) and in posterior mandible augmentation (NCT02745366). Tissue engineered construction based on a synthetic tricalcium phosphate and autologous MSCs obtained from oral mucosa is used for maxilla alveolar process reconstruction in patients with verified diagnosis partially edentulous maxilla and alveolar bone atrophy (NCT02209311). Similarly, MSCs obtained from bone marrow are mixed with bicalcium phosphate to augment the alveolar ridge (NCT02751125).

### Somatic stem cells without scaffolds in oral and maxillofacial regeneration

Stem cells are applied without scaffolds in most clinical trials (32/44 of total trials; Tables 3, 4). There are 5 clinical trials with reported results, 6 clinical trials completed without reported results, and 21 trials ongoing without results. Among the clinical trials with results, the MSCs, PDLSCs, and ADSCs are used.

Oral MSCs have been used to promote the dental pulp and periodontal tissue regeneration. In an ongoing pilot study to determine whether MSC injections directly into mucosal lesions in patients with oral cGVHD are able to alleviate the symptoms and facilitate the reparative process, it states that 55% aGVHD patients who failed front-line steroid treatment responded to MSC infusion in the finished phase II clinical trial (NCT02055625). Intravenously infused allogeneic MSCs suppressed autoimmunity by directing T cells toward Treg and Th2, while suppressing Th17 and Tfh responses, and restored salivary gland secretory function in both mouse models and Sjögren's Syndrome patients who have been resistant to multiple standard treatments (NCT00953485). The study suggests that allogeneic MSC treatment may provide an effective therapy for Sjögren's Syndrome patients (Xu et al., 2012). Although results are not available yet, the other three trials with MSCs focused on osseointegration (NCT02731586), and regeneration of bones (NCT02755922, UMIN000011286).

Human PDLSCs, as well as DPSCs, SHEDs, and bone marrow stem cells have been studied to regenerate periodontal tissues (Seo et al., 2004; Ding et al., 2010; Khorsand et al., 2013; Du et al., 2014; Fu et al., 2014). Clinical trial (NCT01357785) identified no clinical safety problems that could be attributed to the investigational PDLSCs and found a significant increase in the alveolar bone height (decrease in the bone-defect depth) in each group but no statistically significant differences between the PDLSC group and the control group (Chen et al., 2016). Another clinical trial showed reduction of pocket probing depth and clinical attachment gain, while greater linear bone gain at PDLSC sheets/β-TCP treated sites with no adverse reaction in the finished four cases of autologous transplantation of PDLSC sheets to the denuded root surface during the modified Widman flap procedure for the treatment of periodontitis (UMIN000005027, https://www.researchgate.net/publication/266802767). Allogeneic human DPSCs are injected in local infected periodontal tissue for the treatment of chronic periodontal disease (NCT02523651) and a clinical trial UMIN000002050 aims to establish human dental pulp stem cell bank for dental diseases that extraction of tooth is necessary for treatment. A clinical trial is ongoing to clarify the efficiency of autologous SHED to regenerate pulp and periodontal tissue in the patients with immature permanent teeth and pulp necrosis (NCT01814436).

ADSCs represent an attractive and ethical cell source for stem cell therapy and have been used in six clinical trials (Table 3). Intramuscular autologous transplantation of ADSCs enhance the survival of fat grafted into the face in patient with progressive hemifacial atrophy (NCT01309061), suggesting ADSC injection may be used to treat Parry-Romberg disease without the need for microvascular free flap transfer (Koh et al., 2012). The three ongoing clinical trials of ADSCs without scaffolds aim to treat periodontitis, radiation induced xerostomia, and cranial nerve dysfunction.

## Application of oral derived stem cells in non-oral diseases

Oral derived multipotent mesenchymal stem cells are able to differentiate into odontoblast, cementoblast, osteoblast, chondrocyte, adipocyte, melanocyte, endothelial cell hepatocyte, and even myoblast and neural cell. Hence, besides the area of oral diseases, oral derived stem cells have the potential to be applied in brain, eye, heart, liver, bone, skin, and muscle diseases as well (Liu et al., 2015). In terms of animal models, researcher from Japan (Mita et al., 2015) reported that intranasal administration of SHED in a mouse model of Alzheimer's disease (AD) could result in substantially improved cognitive function. The conditioned medium of SHED containing the factors involved in multiple neuro-regenerative mechanisms might account for the results. Iran scientists also showed that improvement of behavioral symptoms was efficiently observed in the mouse model of local sciatic demyelination damage by lysolecithin after transplantation of the DPSCs, to which a tetracycline (Tet)-inducible system expressing OLIG2 gene had been transfected (Askari et al., 2015). And Maraldi et al. confirmed strong potential of bioengineered constructs of stem cell (including dental pulp derived stem cell)-collagen scaffold for correcting large cranial defects in a rat model and highlighting the role of stem cells in neovascularization during skeletal defect reconstruction (Maraldi et al., 2013). Factors secreted from dental pulp stem cells also showed multifaceted benefits for treating experimental rheumatoid arthritis in animal models (Ishikawa et al., 2016). As it was significantly effective both in animal models. Evidence has been proved to be sufficient to step forward from preclinical animal models to human studies in regenerative medicine using oral-derived stem cells. Clinicians and researchers from Japan, Mainland China, Taiwa, South Korea, and France have all carried out clinical trials in eye diseases (Table 4). Oral mucosal epithelial cell sheet was used as the source of stem cells, and they were applied to treat keratoconjunctival disease, corneal epithelial stem cell deficiency, and limbal stem cell deficiency. As most of the trials are under the condition of recruiting, no results are posted online.

## Application of iPS cells in dentistry

### iPS cell generation from fibroblasts with four defined factors

For the application of iPS cells in disease treatment, the first step is to get iPS cells with defined factors. Four factors Oct4, Sox2, Nanog, and Lin28 are sufficient to induce pluripotent stem cells from neonate fibroblasts (Yu et al., 2007). Masato and colleagues found that some family proteins of the four factors Oct3/4, Sox2, Klf4, and c-Myc are capable to reprogram mouse embryonic fibroblasts (MEFs) to iPS cells, and they also found that iPS cells can be generated without Myc although the generation of iPS cells takes more time than with Myc (Nakagawa et al., 2008). More than that, it has been shown that iPS cells can be induced from primary human fibroblasts with Oct4 and Sox2 or be acquired from mouse neural stem cells by introducing only one factor, Oct3/4 (Huangfu et al., 2008; Kim et al., 2009). Nevertheless, the four factors play important roles in the reprogramming, but unique factors may be needed for different cell type.

### Generation of dental stem cells with iPS cell technology

Four factors Oct4, Sox2, Nanog, and Lin28 or Oct3/4, Sox2, Klf4, and c-Myc carried by viral vectors can reprogram three different dental stem cells: SHED, SCAP, and DPSCs (Yan et al., 2010). It has also been demonstrated that three factors without c-Myc can reprogram adult wild-type mouse gingival fibroblasts (GFs), but generation of iPS cells from primary human gingival fibroblasts need four factors (Egusa et al., 2010; Wada et al., 2011). In dentistry, researchers prefer to adopt the classical Yamanaka iPS cell method. But dental tissues are very different from fibroblasts, and there may be new transcription factors involved in the induction. It was shown that miRNA-720 expressed in DPSCs participates in the regulation of the pluripotency factor Nanog and was involved in reprogramming (Hara et al., 2013; Eguchi and Kuboki, 2016). MSC-like populations are rich in dental tissues, which suggest that dental tissues have great potential to be directly reprogrammed into other tissues using defined transcription factors cultured in specific condition. More studies are needed to optimize iPS cell method for dental tissue reprogramming.

### Dental tissue regeneration with iPS cells

Many endeavors have been made with iPS cells to produce new cementum, alveolar bone, ameloblast, and so on (Figure 3; Duan et al., 2011; Yoshida et al., 2015). More and more researchers are interested in the possibility of tooth regeneration from iPS cells. The first trial to generate tooth-like structure from iPS derived neural crest like cells was failed (Otsu et al., 2012). Shortly, tooth-like structures, with bone-like and dental pulp-like areas, were generated from mouse iPS cells by mixing iPS cells with both embryonic dental epithelial and mesenchymal cells (Wen et al., 2012). Furthermore, it was shown that tooth-like structures, of which chemical composition are similar with human teeth, can be generated from patient's own urine induced pluripotent stem cells (Cai et al., 2013). But generation of whole teeth from iPS cells is still a challenge (Wen et al., 2012). The formation of a tooth germ need the interaction between ectoderm-derived epithelial cells and neural-crest-derived mesenchymal cells, so iPS cells are supposed to differentiate into two lineages, and one of them must be odontogenic (Ning et al., 2010). However, it was showed that ES cell or iPS cell derived dental epithelial cells did not have odontogenic potential (Arakaki et al., 2012). Although, mouse iPS cells have potential to differentiate into odontogenic mesenchymal cells through neural crest-like cells, but no signal of tooth generation was shown (Otsu et al., 2012). More work should be done to regenerate a whole tooth.

### Challenges for the application of iPS cells in dentistry

Using retroviruses to generate iPS cells is simple and reproducible, and is the first choice *in vitro* studies. Integrating viruses such as retroviruses or lentiviruses to deliver transcription factors may cause insertional mutagenesis and induce tumorigenesis. New techniques including transduction of plasmids, proteins, and episomal expression vectors which will disappear during culture are emerging (Okita et al., 2008; Eggenschwiler and Cantz, 2009; Yu et al., 2009; Zhou et al., 2009). However, virus-free methods are far less effective. In dentistry, retroviruses and plasmids are favored methods. For clinical application, integration-free methods are essential and more effective integration-free methods are needed.

It was shown that 30% for eight different human iPS cell lines differentiated into tooth-like structures successfully, although it was less efficient compared with 100% differentiation efficiency of mouse embryonic dental epithelium and dental mesenchyme (Cai et al., 2013). It has been questioned always that the iPS cells showed poorer differentiation capacity than ESCs. Neural differentiation of human iPS cells showed lower efficiency than ESCs (Hu et al., 2010) while other groups demonstrated that the ability of human iPS cells to differentiate into motor neurons has no difference with ESCs (Boulting et al., 2011). Although there must be inherent differences among ESCs, MSCs, and iPS cells, culture condition including types of feeder cells, medium, etc. are equally important to successful differentiation. All the conditions should be taken into account to optimize method. Ten years of iPS cell studies are full of surprises and challenges. Tooth regeneration was considered to be an important test ground for application of iPS cells and will meet new surprises and challenges.

## Perspectives

**Figure 4:**
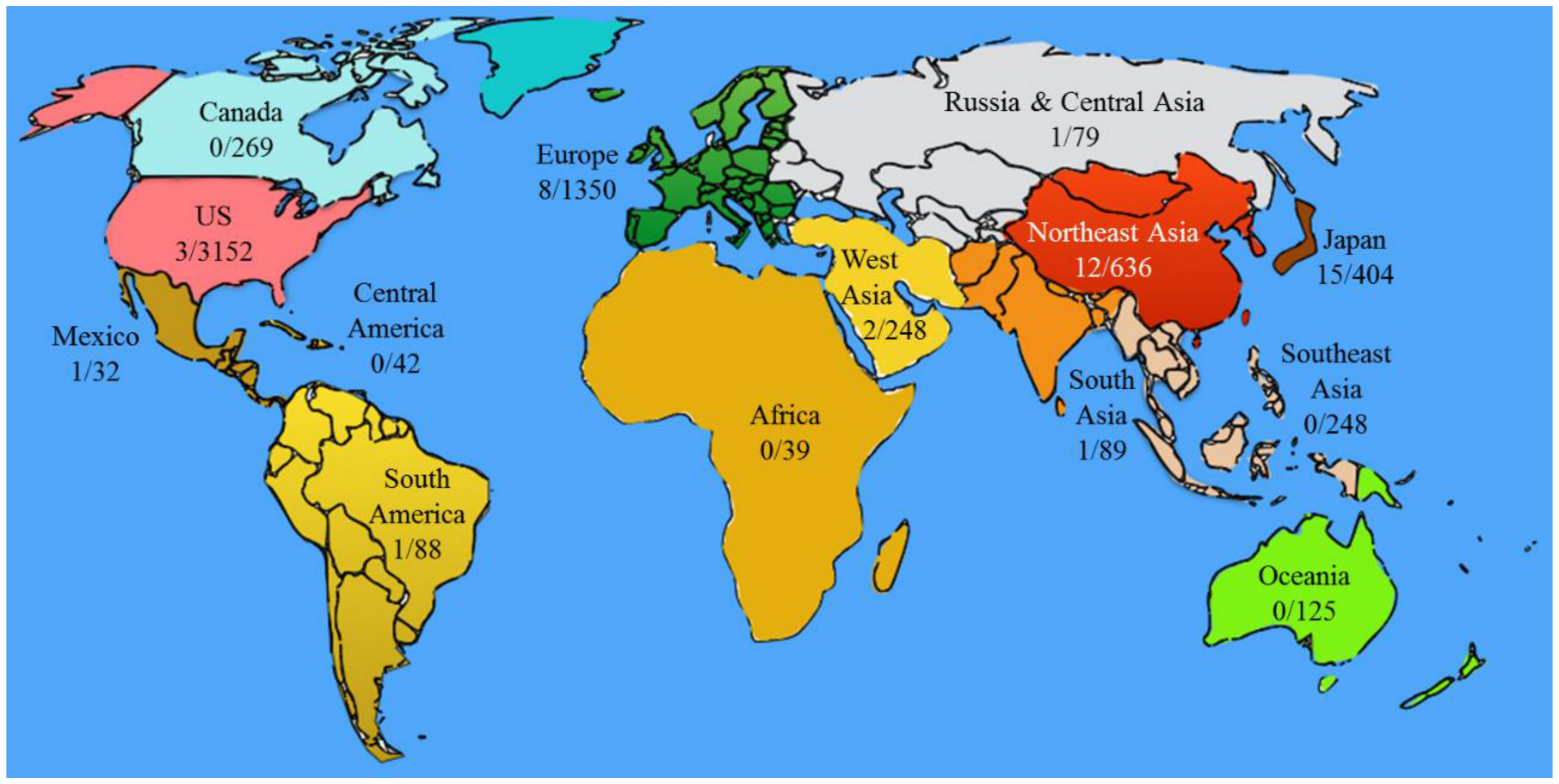
**Clinical trials correlated with oral stem cell and oral diseases in different regions**. The numbers represent the number of clinical trials (oral-related/total stem cell). The “oral-related” cases were generated as same as Table 3. The numbers of “total stem cell” are the summary of the cases obtained by searching “stem cell” on clinicaltrials.gov and umin.ac.jp. Regional division was according to that on clinicaltrials.gov.

There are more than 6,000 clinical trials correlated with stem cells but only total 44 registered clinical trials correlated with oral disease and oral stem cells worldwide (Figure 4, Tables 3, 4, more details could be found in Supplementary Table 1). Most of them are not completed and thus do not provide results. Homogeneity of stem cells, their delivery methods, the quality of regenerated tissues and whether it could be integrated with the host are still the problems to be solved (Mitsiadis et al., 2015). There are two main obstacles in stem cell therapy especially in tissue repair and regeneration. One is how to get enough desirable stem cells, the other is how to direct the differentiation of stem cells into functional cells and tissues. To get enough desirable adult stem cells, accurate cell surface markers should be screened or identified and then they could be used for isolation and enrichment of the stem cells. The next important step is to set up an optimized condition that the isolated stem cells could proliferate and expand to required amount but not differentiate into any other cells. For iPS cells, it is a key step to set up conditions to induce desirable pluripotent cells. To achieve these goals, stem cell fate should be under control.

### Epigenetic modification or interference is the main strategy to control stem cell fate

Epigenetic modifications including modification of histone, DNA methylation, and regulation of micro RNAs are established to affect the maintenance and differentiation of stem cells. DNA methylation and histone modifications are two primary mechanisms to regulate transcriptions of genes in adult stem cells. Generally, DNA methylation of CpG is associated with transcriptional repression, while DNA demethylation activate gene transcription. The modifications of post-translational histone including methylation and acetylation are major epigenetic regulation in dental pulp stem cells. The functions of miRNAs in epigenetic regulation of stem cells are extensively studied recently. Regulation of miRNA mainly rely on translational inhibition by degradation of targeted mRNA. Several miRNAs such as miRNA-204/211, miR-138, miR-125b, and miR-21 are important regulators involved in the osteogenic or adipogenic differentiation of bone marrow stem cells (BMSCs; Eskildsen et al., 2011; Lu et al., 2013; Hu et al., 2014; Wu et al., 2015). miR-720 was observed to promote odontogenic differentiation in dental pulp stem cells (Hara et al., 2013). Alteration of epigenetic modifications by inhibitors can affect the reprogramming of somatic cells or stem cells fate. It has been demonstrated that inhibition of DNA methyltransferase can increase the reprogramming efficiency of somatic cells dramatically (He et al., 2010). The balance between acetylation and deacetylation can be altered by histone deacetylases inhibitors (HDACi). HDACi including trichostatin A (TSA) and valproic acid VPA can promote proliferation and odontoblast differentiation of hDPSCs (Jin et al., 2013; Paino et al., 2014; Duncan et al., 2016).

Growing evidence shows that epigenetic modifications play a crucial role in the induction of iPS cell. As well as four defined factors, reprogramming of histone modifications are required for iPS cell induction. The situation of histone modification in iPS cells and ESCs in pluripotency associated genes are in the same, but in contrast to the differentiated cells. However, the epigenetic reprogramming in iPS cells is not complete since numerous differences in DNA methylation between human ESCs and iPS cells were observed (Lister et al., 2011). These differences are generated by reprogramming as well as epigenetic memories from origin tissues, and will affect the differentiation and application of iPS cells (Kim et al., 2010). For few knowledge about the epigenetic differences between iPS cells and ESCs, complete reprogramming can't be achieved now. Further efforts should be drawn to this field.

### The combination of 3D printing, nanotechnology, and biomedicine is the main state of the art technology to precisely direct stem cell differentiation *in vivo*

Because of the progresses made recently, it is expected to be no problem to get enough desirable stem cells in near future. The most challenging issue is how to precisely control the differentiation of adult stem cells and iPS cells *in vitro* and *in vivo*. To address the issues, the researches could focus on three directions. One is uncovering the differentiation pathways of stem cells to a specific cell type. This step is most important and difficult. The second is to precisely drive the differentiation of stem cells *in vitro* with the target molecules, cytokines, or transcription factors identified in the clarified differentiation pathways. This step can also check or update the previous findings. The last one is controlling stem cells to develop accurately *in vivo*. This step, unlike the *in vitro* differentiation, is very complicated due to the body responses and undefined micro-environment. One ignored issue in wound repair and organ regeneration with stem cells is that the stem cells may migrate from the target area to elsewhere, thus reducing the therapy efficiency. Moreover, the cytokines or differentiating factors used to control stem cell fate will distribute everywhere in the body, which exaggerate the difficulty of precisely direct stem cell differentiation. The 3D print technology and nanotechnology may help solve these issues.

Nanotechnology is defined as the manipulation of matter with at least one dimension sized from 1 to 100 nanometers. Nanomaterials have unique optical, electronic, or mechanical properties and thus show special bioeffects. In a recent study with DPSCs, the ROS-scavenging events of cerium nanomaterials (CeNMs) were related to the aspect ratio-dependent cellular internalization, suggesting the promising use of CeNMs to protect stem cells from the ROS-insult environments and ultimately improve the stem cell potential for tissue engineering and regenerative medicine (Mahapatra et al., 2017). In another study, it is reported that enhanced rapid bone regeneration and complete mature bone-structure formation was obtained when using the physiological electric potential and the fabricated nanocomposite membrane mimicking the endogenous electric potential (Zhang et al., 2016). It provides another strategy to compose novel scaffolds for dental stem cells. And Heo et al. found that gold nanoparticles (GNPs) coated titanium could enhance the osteogenic differentiation in human adipose-derived stem cells (Heo et al., 2016). What's more, nano-thin polymeric shells constructed and modified by nanostructure involvement were used to immobilize the DPSCs with a layer-by-layer technique to ensure the layers stability and integrity as well as separation from bacterial cells (Grzeczkowicz et al., 2015).

**Figure 5:**
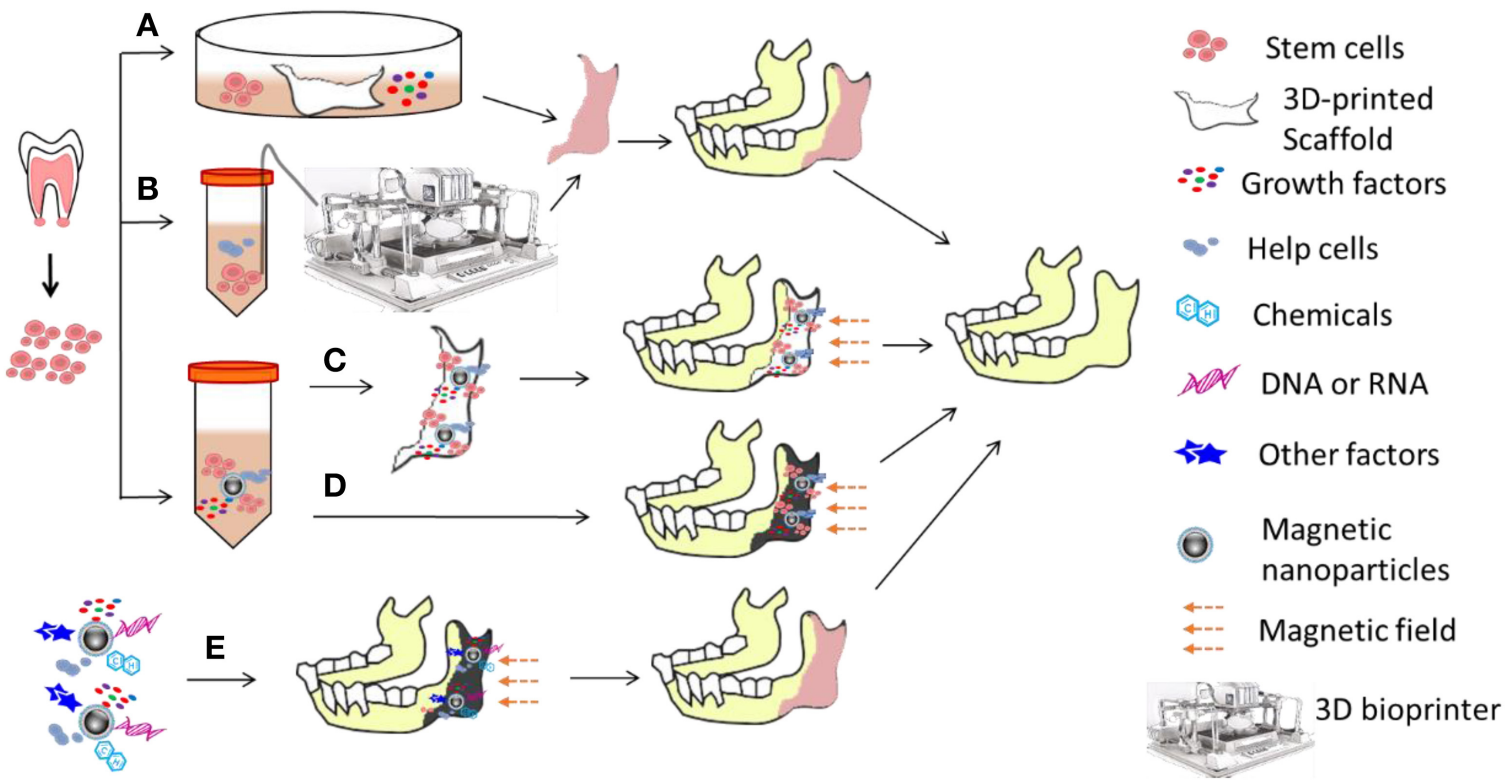
**Strategies for oral disease treatment with stem cells**. Oral stem cells are sorted out and expanded *in vitro* and then **(A)** co-cultured with or seeded in a 3D-printed scaffold made of nanomaterials in medium with growth factors for hours or days to form an organ-like structure, or **(B)** mixed with biomaterials, helper cells, and growth factors to make bioink for bioprinters to print an organ-like structure which is immediately used for repair and reconstruction of impaired bones. Together with helper cells and growth factors, stem cells can also be linked to nanomaterials (here showing magnetic nanoparticles) to form an immobilized format (changed from free moving format) which can be loaded/seeded in either **(C)** the scaffold which will be implanted to impaired region, or **(D)** directly in the impaired region. Magnetic field will prevent the migration of cells and growth factors/cytokines, and thus form a relative steady microenvironment which could direct stem cell differentiation. **(E)** *In situ* stem cells around the impaired region could also be attracted and/or stimulated for controlled differentiation in the artificial microenvironment which is established with magnetic immobilized helper cells, bioactive factors, gene regulation molecules, and chemicals.

3D bioprinting, the combination of 3D printing technology, biology, and materials science, is the process of direct printing of biological materials (cells, proteins, etc.) into a confined space, where biological function and viability of biomaterials are preserved within the printed construct (Murphy and Atala, 2014). One of the three major concepts in 3D bioprinting is the type of bio-ink, which is made up of poly (ethylene glycol; Arcaute et al., 2006; Chan et al., 2010), fibrinogen (Cui and Boland, 2009; Lee et al., 2009) and/or alginate (Fedorovich et al., 2012; Hockaday et al., 2012) and helped to maintain both the biological function and viability of biomaterials and the dimensions of printed objects (Shafiee and Atala, 2016). The combination of nanotechnology and 3D printing technology, allows the creation of sophisticated materials with exquisite fine structural detail and provides fantastic therapy strategies. Figure 5 outlines the strategies used potentially in oral disease treatment. For example, bone stem cells can be administrated in a 3D printed biocompatible and biodegradable scaffold which is made of nanomaterials and matches the bone wound. Stem cells, cytokines and cytokine expressing plasmids can be either used as bioink to directly print an organ-like structure with a bioprinter or loaded on magnetic nanoparticles (Sun et al., 2011) and then precisely immobilized in the target area with magnetic field. The design of novel nanostructured materials, such as biomimetic matrices and scaffolds for controlling cell fate and differentiation, and nanoparticles for diagnostics, imaging and targeted treatment, is a novel direction for stem cell therapy. The administration to patients of dynamic biological agents comprising stem cells, bioactive scaffolds and/or nanoparticles will certainly increase the regenerative impact of dental pathological tissues (Mitsiadis et al., 2012).

### Biosafety and the development of technology determine which kind of stem cells to be used for oral disease treatment

In summary, there are endogenous/autologous and exogenous/allogeneic adult stem cells and iPS cells for oral disease treatment. As compared in Table 5, these cells have their own pros and cons features. The features suggest the main research direction of stem cell therapy. For autologous/endogenous stem cells, there are two main research directions: try to expand the stem cells *in vitro* without stemness loss or function change; try to speed up self-renewal of the *in situ* stem cells and precisely control their differentiation. For allogeneic stem cells, the *in vitro* expand technology is also very important and the technology to lower down the immune rejection is urgently needed. Besides the immune suppression drugs, regulatory immune cells such as Treg and Breg (Sun et al., 2015; Xu et al., 2016) may be used to overcome the immune rejection and enhance the efficiency of stem cell therapy. For iPS cells, the biosafety is the main issue that should be addressed due to their high tumorigenesis. The advances of technologies in these directions determine which stem cells could easily reach a desirable number and show highest biosafety and lowest immunogenicity, and therefore would be clinically used successfully.

**Table 5:** **Pros and cons of stem cells with different origins in clinical application**.

	**Autologous stem cells**	**Allogeneic stem cells**	**iPS cells**	**References**
Pros	Hypoimmunogenic or non-immunogenic; some of them display immunomodulatory; derived from accessible tissues such as human exfoliated deciduous teeth	Derived from healthy tissues; with numerous clinical studies; an established treatment strategy for many malignant hematological diseases	Pluripotency; non-immunogenic; unlimited source; no ethical issues;	Girlovanu et al., 2015; Juric et al., 2016
Cons	Limited differentiation potential and finite life span; limited sources; difficult to treat the disease resulted from gene mutations	Ethical problems; limited sources (lack of suitable donor organs and tissues); immune rejection	Differentiation potential limited by epigenetic memory; tumorigenesis	Consentius et al., 2015; Girlovanu et al., 2015

## Author contributions

BY, YQ, and NZ summarized the literature, wrote the manuscript, and prepared figures. BC, HO, and JD provided critical comments and wrote part of the manuscript. JS supervised all the works and wrote the manuscript.

### Conflict of interest statement

The authors declare that the research was conducted in the absence of any commercial or financial relationships that could be construed as a potential conflict of interest. The reviewer VD, VT and handling Editor declared their shared affiliation, and the handling Editor states that the process nevertheless met the standards of a fair and objective review.

## References

[B1] Arakaki M. Egusa H. Otsu K. Saitoh K. Miura T. Harada H. (2013). Frontier dental research on iPS cells. J. Oral Biosci. 55, 191–199. 10.1016/j.job.2013.08.002

[B2] Arakaki M. Ishikawa M. Nakamura T. Iwamoto T. Yamada A. Fukumoto E.. (2012). Role of epithelial-stem cell interactions during dental cell differentiation. J. Biol. Chem. 287, 10590–10601. 10.1074/jbc.M111.285874 22298769 PMC3323010

[B3] Arcaute K. Mann B. K. Wicker R. B. (2006). Stereolithography of three-dimensional bioactive poly(ethylene glycol) constructs with encapsulated cells. Ann. Biomed. Eng. 34, 1429–1441. 10.1007/s10439-006-9156-y 16897421

[B4] Askari N. Yaghoobi M. M. Shamsara M. Esmaeili-Mahani S. (2015). Tetracycline-regulated expression of OLIG2 gene in human dental pulp stem cells lead to mouse sciatic nerve regeneration upon transplantation. Neuroscience 305, 197–208. 10.1016/j.neuroscience.2015.07.088 26254831

[B5] Baba S. Yamada Y. (2016). Phase I/II Trial of autologous bone marrow stem cell transplantation with a three-dimensional woven-fabric scaffold for periodontitis. Stem Cells Int. 2016:6205910. 10.1155/2016/6205910 27990164 PMC5136404

[B6] Bakhtiar H. Mirzaei H. Bagheri M. R. Fani N. Mashhadiabbas F. Baghaban Eslaminejad M.. (2016). Histologic tissue response to furcation perforation repair using mineral trioxide aggregate or dental pulp stem cells loaded onto treated dentin matrix or tricalcium phosphate. Clin. Oral Investig. [Epub ahead of print]. 10.1007/s00784-016-1967-0 27761672 PMC5442265

[B7] Bhuptani R. S. Patravale V. B. (2016). Porous microscaffolds for 3D culture of dental pulp mesenchymal stem cells. Int. J. Pharm. 515, 555–564. 10.1016/j.ijpharm.2016.10.040 27989823

[B8] Boulting G. L. Kiskinis E. Croft G. F. Amoroso M. W. Oakley D. H. Wainger B. J.. (2011). A functionally characterized test set of human induced pluripotent stem cells. Nat. Biotechnol. 29, 279–286. 10.1038/nbt.1783 21293464 PMC3229307

[B9] Cai J. Zhang Y. Liu P. Chen S. Wu X. Sun Y.. (2013). Generation of tooth-like structures from integration-free human urine induced pluripotent stem cells. Cell Regen. 2:6. 10.1186/2045-9769-2-6 25408878 PMC4230506

[B10] Caplan A. I. (1991). Mesenchymal stem cells. J. Orthop. Res. 9, 641–650. 10.1002/jor.1100090504 1870029

[B11] Caton J. Bostanci N. Remboutsika E. De Bari C. Mitsiadis T. A. (2011). Future dentistry: cell therapy meets tooth and periodontal repair and regeneration. J. Cell. Mol. Med. 15, 1054–1065. 10.1111/j.1582-4934.2010.01251.x 21199329 PMC3822618

[B12] Chamieh F. Collignon A. M. Coyac B. R. Lesieur J. Ribes S. Sadoine J.. (2016). Accelerated craniofacial bone regeneration through dense collagen gel scaffolds seeded with dental pulp stem cells. Sci. Rep. 6:38814. 10.1038/srep38814 27934940 PMC5146967

[B13] Chan V. Zorlutuna P. Jeong J. H. Kong H. Bashir R. (2010). Three-dimensional photopatterning of hydrogels using stereolithography for long-term cell encapsulation. Lab Chip 10, 2062–2070. 10.1039/c004285d 20603661

[B14] Chen F. M. Gao L. N. Tian B. M. Zhang X. Y. Zhang Y. J. Dong G. Y.. (2016). Treatment of periodontal intrabony defects using autologous periodontal ligament stem cells: a randomized clinical trial. Stem Cell Res. Ther. 7, 33. 10.1186/s13287-016-0288-1 26895633 PMC4761216

[B15] Cho H. Tarafder S. Fogge M. Kao K. Lee C. H. (2016). Periodontal ligament stem/progenitor cells with protein-releasing scaffolds for cementum formation and integration on dentin surface. Connect. Tissue Res. 57, 488–495. 10.1080/03008207.2016.1191478 27215800

[B16] Chrepa V. Henry M. A. Daniel B. J. Diogenes A. (2015). Delivery of apical mesenchymal stem cells into root canals of mature teeth. J. Dent. Res. 94, 1653–1659. 10.1177/0022034515596527 26195498 PMC6728573

[B17] Consentius C. Reinke P. Volk H. D. (2015). Immunogenicity of allogeneic mesenchymal stromal cells: what has been seen *in vitro* and *in vivo*? Regen. Med. 10, 305–315. 10.2217/rme.15.14 25933239

[B18] Cui X. Boland T. (2009). Human microvasculature fabrication using thermal inkjet printing technology. Biomaterials 30, 6221–6227. 10.1016/j.biomaterials.2009.07.056 19695697

[B19] d'Aquino R. De Rosa A. Laino G. Caruso F. Guida L. Rullo R.. (2009b). Human dental pulp stem cells: from biology to clinical applications. J. Exp. Zool. B Mol. Dev. Evol. 312B, 408–415. 10.1002/jez.b.21263 19065566

[B20] d'Aquino R. De Rosa A. Lanza V. Tirino V. Laino L. Graziano A.. (2009a). Human mandible bone defect repair by the grafting of dental pulp stem/progenitor cells and collagen sponge biocomplexes. Eur. Cell. Mater. 18, 75–83. 10.22203/eCM.v018a07 19908196

[B21] Ding G. Liu Y. Wang W. Wei F. Liu D. Fan Z.. (2010). Allogeneic periodontal ligament stem cell therapy for periodontitis in swine. Stem Cells 28, 1829–1838. 10.1002/stem.512 20979138 PMC2996858

[B22] Du J. Shan Z. Ma P. Wang S. Fan Z. (2014). Allogeneic bone marrow mesenchymal stem cell transplantation for periodontal regeneration. J. Dent. Res. 93, 183–188. 10.1177/0022034513513026 24226426

[B23] Duan X. Tu Q. Zhang J. Ye J. Sommer C. Mostoslavsky G.. (2011). Application of induced pluripotent stem (iPS) cells in periodontal tissue regeneration. J. Cell. Physiol. 226, 150–157. 10.1002/jcp.22316 20658533 PMC4137963

[B24] Duncan H. F. Smith A. J. Fleming G. J. Cooper P. R. (2016). Epigenetic modulation of dental pulp stem cells: implications for regenerative endodontics. Int. Endod. J. 49, 431–446. 10.1111/iej.12475 26011759

[B25] Eggenschwiler R. Cantz T. (2009). Induced pluripotent stem cells generated without viral integration. Hepatology 49, 1048–1049. 10.1002/hep.22827 19242974

[B26] Eguchi T. Kuboki T. (2016). Cellular reprogramming using defined factors and MicroRNAs. Stem Cells Int. 2016:7530942. 10.1155/2016/7530942 27382371 PMC4921148

[B27] Egusa H. Okita K. Kayashima H. Yu G. Fukuyasu S. Saeki M.. (2010). Gingival fibroblasts as a promising source of induced pluripotent stem cells. PLoS ONE 5:e12743. 10.1371/journal.pone.0012743 20856871 PMC2939066

[B28] Egusa H. Sonoyama W. Nishimura M. Atsuta I. Akiyama K. (2012). Stem cells in dentistry–part I: stem cell sources. J. Prosthodont. Res. 56, 151–165. 10.1016/j.jpor.2012.06.001 22796367

[B29] Eskildsen T. Taipaleenmaki H. Stenvang J. Abdallah B. M. Ditzel N. Nossent A. Y.. (2011). MicroRNA-138 regulates osteogenic differentiation of human stromal (mesenchymal) stem cells *in vivo*. Proc. Natl. Acad. Sci. U.S.A. 108, 6139–6144. 10.1073/pnas.1016758108 21444814 PMC3076836

[B30] Fawzy El-Sayed K. M. Dörfer C. E. (2016). Gingival mesenchymal stem/progenitor cells: a unique tissue engineering gem. Stem Cells Int. 2016, 1–16. 10.1155/2016/7154327 27313628 PMC4903147

[B31] Fawzy El-Sayed K. M. Mekhemar M. K. Beck-Broichsitter B. E. Bahr T. Hegab M. Receveur J.. (2015). Periodontal regeneration employing gingival margin-derived stem/progenitor cells in conjunction with IL-1ra-hydrogel synthetic extracellular matrix. J. Clin. Periodontol. 42, 448–457. 10.1111/jcpe.12401 25875208

[B32] Fedorovich N. E. Schuurman W. Wijnberg H. M. Prins H. J. van Weeren P. R. Malda J.. (2012). Biofabrication of osteochondral tissue equivalents by printing topologically defined, cell-laden hydrogel scaffolds. Tissue Eng. Part C Methods 18, 33–44. 10.1089/ten.tec.2011.0060 21854293 PMC3245674

[B33] Feliciano K. M. de Franca Caldas A. Jr. (2006). A systematic review of the diagnostic classifications of traumatic dental injuries. Dent. Traumatol. 22, 71–76. 10.1111/j.1600-9657.2006.00342.x 16499629

[B34] Feng F. Akiyama K. Liu Y. Yamaza T. Wang T. M. Chen J. H.. (2010). Utility of PDL progenitors for *in vivo* tissue regeneration: a report of 3 cases. Oral Dis. 16, 20–28. 10.1111/j.1601-0825.2009.01593.x 20355278 PMC2848819

[B35] Fiedler J. Etzel N. Brenner R. E. (2004). To go or not to go: migration of human mesenchymal progenitor cells stimulated by isoforms of PDGF. J. Cell. Biochem. 93, 990–998. 10.1002/jcb.20219 15389881

[B36] Fu X. Jin L. Ma P. Fan Z. Wang S. (2014). Allogeneic stem cells from deciduous teeth in treatment for periodontitis in miniature swine. J. Periodontol. 85, 845–851. 10.1902/jop.2013.130254 24001042

[B37] Gault P. Black A. Romette J. L. Fuente F. Schroeder K. Thillou F.. (2010). Tissue-engineered ligament: implant constructs for tooth replacement. J. Clin. Periodontol. 37, 750–758. 10.1111/j.1600-051x.2010.01588.x 20546087

[B38] Girlovanu M. Susman S. Soritau O. Rus-Ciuca D. Melincovici C. Constantin A. M.. (2015). Stem cells - biological update and cell therapy progress. Clujul Med. 88, 265–271. 10.15386/cjmed-483 26609255 PMC4632881

[B39] Giuliani A. Manescu A. Langer M. Rustichelli F. Desiderio V. Paino F.. (2013). Three years after transplants in human mandibles, histological and in-line holotomography revealed that stem cells regenerated a compact rather than a spongy bone: biological and clinical implications. Stem Cells Transl. Med. 2, 316–324. 10.5966/sctm.2012-0136 23502599 PMC3659838

[B40] Giuliani A. Manescu A. Mohammadi S. Mazzoni S. Piattelli A. Mangano F.. (2016). Quantitative kinetics evaluation of blocks versus granules of biphasic calcium phosphate scaffolds (HA/β-TCP 30/70) by synchrotron radiation X-ray microtomography. Implant Dent. 25, 6–15. 10.1097/ID.0000000000000363 26630463

[B41] Giuliani A. Moroncini F. Mazzoni S. Belicchi M. L. Villa C. Erratico S.. (2014). Polyglycolic acid-polylactic acid scaffold response to different progenitor cell *in vitro* cultures: a demonstrative and comparative X-ray synchrotron radiation phase-contrast microtomography study. Tissue Eng. Part C Methods 20, 308–316. 10.1089/ten.tec.2013.0213 23879738 PMC3968884

[B42] Grzeczkowicz A. Granicka L. H. Maciejewska I. Strawski M. Szklarczyk M. Borkowska M. (2015). The experimental study of the performance of nano-thin polyelectrolyte shell for dental pulp stem cells immobilization. J. Nanosci. Nanotechnol. 15, 9531–9538. 10.1166/jnn.2015.10840 26682375

[B43] Hara E. S. Ono M. Eguchi T. Kubota S. Pham H. T. Sonoyama W.. (2013). miRNA-720 controls stem cell phenotype, proliferation and differentiation of human dental pulp cells. PLoS ONE 8:e83545. 10.1371/journal.pone.0083545 24386225 PMC3875457

[B44] Hargreaves K. M. Berman L. H. Rotstein I. (2016). Cohen's Pathways of the Pulp, 11th Edn. St. Louis, MO: Elsevier.

[B45] Hayashi Y. Murakami M. Kawamura R. Ishizaka R. Fukuta O. Nakashima M. (2015). CXCL14 and MCP1 are potent trophic factors associated with cell migration and angiogenesis leading to higher regenerative potential of dental pulp side population cells. Stem Cell Res. Ther. 6:111. 10.1186/s13287-015-0088-z 26021377 PMC4488059

[B46] He Z. Liu H. C. Tang Y. Rosenwaks Z. (2010). Enhancing somatic nuclear reprogramming by DNA demethylation with MicroRNA (miRNA) and DNA methyltransferase knockdown. Fertil. Steril. 94:S56 10.1016/j.fertnstert.2010.07.218

[B47] Heo D. N. Ko W. K. Lee H. R. Lee S. J. Lee D. Um S. H.. (2016). Titanium dental implants surface-immobilized with gold nanoparticles as osteoinductive agents for rapid osseointegration. J. Colloid Interface Sci. 469, 129–137. 10.1016/j.jcis.2016.02.022 26874978

[B48] Hockaday L. A. Kang K. H. Colangelo N. W. Cheung P. Y. C. Duan B. Malone E.. (2012). Rapid 3D printing of anatomically accurate and mechanically heterogeneous aortic valve hydrogel scaffolds. Biofabrication 4:035005. 10.1088/1758-5082/4/3/035005 22914604 PMC3676672

[B49] Horst O. V. Chavez M. G. Jheon A. H. Desai T. Klein O. D. (2012). Stem cell and biomaterials research in dental tissue engineering and regeneration. Dent. Clin. North Am. 56, 495–520. 10.1016/j.cden.2012.05.009 22835534 PMC3494412

[B50] Hu B. Y. Weick J. P. Yu J. Ma L. X. Zhang X. Q. Thomson J. A.. (2010). Neural differentiation of human induced pluripotent stem cells follows developmental principles but with variable potency. Proc. Natl. Acad. Sci. U.S.A. 107, 4335–4340. 10.1073/pnas.0910012107 20160098 PMC2840097

[B51] Hu X. Y. Chen P. Wu Y. Zhu W. Wang J. (2014). Abstract 249: Mir-211 regulates bone marrow mesenchymal stem cells migration through Stat5a. Circ. Res. 115(Suppl. 1), A249–A249.

[B52] Huang P. He Z. Ji S. Sun H. Xiang D. Liu C.. (2011). Induction of functional hepatocyte-like cells from mouse fibroblasts by defined factors. Nature 475, 386–389. 10.1038/nature10116 21562492

[B53] Huangfu D. Osafune K. Maehr R. Guo W. Eijkelenboom A. Chen S.. (2008). Induction of pluripotent stem cells from primary human fibroblasts with only Oct4 and Sox2. Nat. Biotechnol. 26, 1269–1275. 10.1038/nbt.1502 18849973

[B54] Ishikawa J. Takahashi N. Matsumoto T. Yoshioka Y. Yamamoto N. Nishikawa M.. (2016). Factors secreted from dental pulp stem cells show multifaceted benefits for treating experimental rheumatoid arthritis. Bone 83, 210–219. 10.1016/j.bone.2015.11.012 26603475

[B55] Jin H. Park J. Y. Choi H. Choung P. H. (2013). HDAC inhibitor trichostatin A promotes proliferation and odontoblast differentiation of human dental pulp stem cells. Tissue Eng. Part A 19, 613–624. 10.1089/ten.tea.2012.0163 23013422

[B56] Juric M. K. Ghimire S. Ogonek J. Weissinger E. M. Holler E. van Rood J. J.. (2016). Milestones of hematopoietic stem cell transplantation - from first human studies to current developments. Front. Immunol. 7:470. 10.3389/fimmu.2016.00470 27881982 PMC5101209

[B57] Karagiannis P. Eto K. (2016). Ten years of induced pluripotency: from basic mechanisms to therapeutic applications. Development 143, 2039–2043. 10.1242/dev.138172 27302394

[B58] Kemoun P. Laurencin-Dalicieux S. Rue J. Farges J. C. Gennero I. Conte-Auriol F.. (2007). Human dental follicle cells acquire cementoblast features under stimulation by BMP-2/-7 and enamel matrix derivatives (EMD) *in vitro*. Cell Tissue Res. 329, 283–294. 10.1007/s00441-007-0397-3 17443352

[B59] Khorsand A. Eslaminejad M. B. Arabsolghar M. Paknejad M. Ghaedi B. Rokn A. R.. (2013). Autologous dental pulp stem cells in regeneration of defect created in canine periodontal tissue. J. Oral Implantol. 39, 433–443. 10.1563/AAID-JOI-D-12-00027 23964777

[B60] Kim B. C. Jun S. M. Kim S. Y. Kwon Y. D. Choe S. C. Kim E. C.. (2016). Engineering three dimensional micro nerve tissue using postnatal stem cells from human dental apical papilla. Biotechnol. Bioeng. 114, 903–914. 10.1002/bit.26205 27775170

[B61] Kim E. Y. Page P. Dellefave-Castillo L. M. McNally E. M. Wyatt E. J. (2016). Direct reprogramming of urine-derived cells with inducible MyoD for modeling human muscle disease. Skelet. Muscle 6, 32. 10.1186/s13395-016-0103-9 27651888 PMC5025576

[B62] Kim J. B. Sebastiano V. Wu G. Arauzo-Bravo M. J. Sasse P. Gentile L.. (2009). Oct4-induced pluripotency in adult neural stem cells. Cell 136, 411–419. 10.1016/j.cell.2009.01.023 19203577

[B63] Kim K. Doi A. Wen B. Ng K. Zhao R. Cahan P.. (2010). Epigenetic memory in induced pluripotent stem cells. Nature 467, 285–290. 10.1038/nature09342 20644535 PMC3150836

[B64] Kitamura M. Akamatsu M. Machigashira M. Hara Y. Sakagami R. Hirofuji T.. (2011). FGF-2 stimulates periodontal regeneration: results of a multi-center randomized clinical trial. J. Dent. Res. 90, 35–40. 10.1177/0022034510384616 21059869

[B65] Kitaori T. Ito H. Schwarz E. M. Tsutsumi R. Yoshitomi H. Oishi S.. (2009). Stromal cell-derived factor 1/CXCR4 signaling is critical for the recruitment of mesenchymal stem cells to the fracture site during skeletal repair in a mouse model. Arthritis Rheum. 60, 813–823. 10.1002/art.24330 19248097

[B66] Koh K. S. Oh T. S. Kim H. Chung I. W. Lee K. W. Lee H. B.. (2012). Clinical application of human adipose tissue-derived mesenchymal stem cells in progressive hemifacial atrophy (Parry-Romberg disease) with microfat grafting techniques using 3-dimensional computed tomography and 3-dimensional camera. Ann. Plast. Surg. 69, 331–337. 10.1097/SAP.0b013e31826239f0 22907186

[B67] Krebsbach P. H. Kuznetsov S. A. Satomura K. Emmons R. V. Rowe D. W. Robey P. G. (1997). Bone formation *in vivo*: comparison of osteogenesis by transplanted mouse and human marrow stromal fibroblasts. Transplantation 63, 1059–1069. 10.1097/00007890-199704270-00003 9133465

[B68] Lee C. H. Cook J. L. Mendelson A. Moioli E. K. Yao H. Mao J. J. (2010). Regeneration of the articular surface of the rabbit synovial joint by cell homing: a proof of concept study. Lancet 376, 440–448. 10.1016/S0140-6736(10)60668-X 20692530 PMC4035014

[B69] Lee W. Debasitis J. C. Lee V. K. Lee J. H. Fischer K. Edminster K.. (2009). Multi-layered culture of human skin fibroblasts and keratinocytes through three-dimensional freeform fabrication. Biomaterials 30, 1587–1595. 10.1016/j.biomaterials.2008.12.009 19108884

[B70] Liao J. Al Shahrani M. Al-Habib M. Tanaka T. Huang G. T. (2011). Cells isolated from inflamed periapical tissue express mesenchymal stem cell markers and are highly osteogenic. J. Endod. 37, 1217–1224. 10.1016/j.joen.2011.05.022 21846537 PMC3499979

[B71] Lima R. L. Holanda-Afonso R. C. Moura-Neto V. Bolognese A. M. DosSantos M. F. Souza M. M. (2017). Human dental follicle cells express embryonic, mesenchymal and neural stem cells markers. Arch. Oral Biol. 73, 121–128. 10.1016/j.archoralbio.2016.10.003 27764680

[B72] Lister R. Pelizzola M. Kida Y. S. Hawkins R. D. Nery J. R. Hon G.. (2011). Hotspots of aberrant epigenomic reprogramming in human induced pluripotent stem cells. Nature 471, 68–73. 10.1038/nature09798 21289626 PMC3100360

[B73] Liu J. Yu F. Sun Y. Jiang B. Zhang W. Yang J.. (2015). Concise reviews: characteristics and potential applications of human dental tissue-derived mesenchymal stem cells. Stem Cells 33, 627–638. 10.1002/stem.1909 25447379

[B74] Lu X. Deng M. He H. Zeng D. Zhang W. (2013). [miR-125b regulates osteogenic differentiation of human bone marrow mesenchymal stem cells by targeting Smad4]. Zhong Nan Da Xue Xue Bao Yi Xue Ban 38, 341–346. 10.3969/j.issn.1672-7347.2013.04.002 23645233

[B75] Lucaciu O. Soriţău O. Gheban D. Ciuca D. R. Virtic O. Vulpoi A.. (2015). Dental follicle stem cells in bone regeneration on titanium implants. BMC Biotechnol. 15:6. 10.1186/s12896-015-0229-6 26718927 PMC4697321

[B76] Luu H. H. Song W. X. Luo X. Manning D. Luo J. Deng Z. L.. (2007). Distinct roles of bone morphogenetic proteins in osteogenic differentiation of mesenchymal stem cells. J. Orthop. Res. 25, 665–677. 10.1002/jor.20359 17290432

[B77] Ma G. Zhao J. L. Mao M. Chen J. Dong Z. W. Liu Y. P. (2016). Scaffold-based delivery of bone marrow mesenchymal stem cell sheet fragments enhances new bone formation *in vivo*. J. Oral Maxillofac. Surg. 75, 92–104. 10.1016/j.joms.2016.08.014 27637777

[B78] Mahapatra C. Singh R. K. Lee J. H. Jung J. Keun Hyun J. Kim H. W. (2017). Nano-shape varied cerium oxide nanomaterials rescue human dental stem cells from oxidative insult through intracellular or extracellular actions. Acta Biomater. 50, 142–153. 10.1016/j.actbio.2016.12.014 27940193

[B79] Mangano C. De Rosa A. Desiderio V. d'Aquino R. Piattelli A. De Francesco F.. (2010). The osteoblastic differentiation of dental pulp stem cells and bone formation on different titanium surface textures. Biomaterials 31, 3543–3551. 10.1016/j.biomaterials.2010.01.056 20122719

[B80] Mangano C. Paino F. d'Aquino R. De Rosa A. Iezzi G. Piattelli A.. (2011). Human dental pulp stem cells hook into biocoral scaffold forming an engineered biocomplex. PLoS ONE 6:4. 10.1371/journal.pone.0018721 21494568 PMC3073992

[B81] Mao J. J. Prockop D. J. (2012). Stem cells in the face: tooth regeneration and beyond. Cell Stem Cell 11, 291–301. 10.1016/j.stem.2012.08.010 22958928 PMC4093804

[B82] Maraldi T. Riccio M. Pisciotta A. Zavatti M. Carnevale G. Beretti F.. (2013). Human amniotic fluid-derived and dental pulp-derived stem cells seeded into collagen scaffold repair critical-size bone defects promoting vascularization. Stem Cell Res. Ther. 4:53. 10.1186/scrt203 23688855 PMC3706961

[B83] Mari-Beffa M. Segura-Egea J. J. Diaz-Cuenca A. (2017). Regenerative endodontic procedures: a perspective from stem cell niche biology. J. Endod. 43, 52–62. 10.1016/j.joen.2016.09.011 27986102

[B84] Mason C. Dunnill P. (2008). A brief definition of regenerative medicine. Regen. Med. 3, 1–5. 10.2217/17460751.3.1.1 18154457

[B85] Mele L. Vitiello P. P. Tirino V. Paino F. De Rosa A. Liccardo D.. (2016). Changing paradigms in cranio-facial regeneration: current and new strategies for the activation of endogenous stem cells. Front. Physiol. 7:62. 10.3389/fphys.2016.00062 26941656 PMC4764712

[B86] Menabde G. Gogilashvili K. Kakabadze Z. Berishvili E. (2009). Bone marrow-derived mesenchymal stem cell plasticity and their application perspectives. Georgian Med. News 71–76. 19276476

[B87] Menicanin D. Hynes K. Han J. Gronthos S. Bartold P. M. (2015). Cementum and periodontal ligament regeneration. Adv. Exp. Med. Biol. 881, 207–236. 10.1007/978-3-319-22345-2_12 26545752

[B88] Mertens C. Freudlsperger C. Bodem J. Engel M. Hoffmann J. Freier K. (2016). Reconstruction of the maxilla following hemimaxillectomy defects with scapular tip grafts and dental implants. J. Craniomaxillofac. Surg. 44, 1806–1811. 10.1016/j.jcms.2016.08.010 27697398

[B89] Mita T. Furukawa-Hibi Y. Takeuchi H. Hattori H. Yamada K. Hibi H.. (2015). Conditioned medium from the stem cells of human dental pulp improves cognitive function in a mouse model of Alzheimer's disease. Behav. Brain Res. 293, 189–197. 10.1016/j.bbr.2015.07.043 26210934

[B90] Mitsiadis T. A. Orsini G. Jimenez-Rojo L. (2015). Stem cell-based approaches in dentistry. Eur. Cell Mater. 30, 248–257. 10.22203/eCM.v030a17 26562631

[B91] Mitsiadis T. A. Woloszyk A. Jimenez-Rojo L. (2012). Nanodentistry: combining nanostructured materials and stem cells for dental tissue regeneration. Nanomedicine 7, 1743–1753. 10.2217/nnm.12.146 23210714

[B92] Mjor I. A. (2001). Pulp-dentin biology in restorative dentistry. Part 5: clinical management and tissue changes associated with wear and trauma. Quintessence Int. 32, 771–788. 11820046

[B93] Moshaverinia A. Xu X. Chen C. Ansari S. Zadeh H. H. Snead M. L.. (2014). Application of stem cells derived from the periodontal ligament or gingival tissue sources for tendon tissue regeneration. Biomaterials 35, 2642–2650. 10.1016/j.biomaterials.2013.12.053 24397989 PMC3929697

[B94] Motamedian S. R. Tabatabaei F. S. Akhlaghi F. Torshabi M. Gholamin P. Khojasteh A. (2016). Response of dental pulp stem cells to synthetic, Allograft, and Xenograft bone Scaffolds. Int. J. Periodontics Restorative Dent. 37, 47–59. 10.11607/prd.2121 27977818

[B95] Murphy S. V. Atala A. (2014). 3D bioprinting of tissues and organs. Nat. Biotechnol. 32, 773–785. 10.1038/nbt.2958 25093879

[B96] Murray P. E. Garcia-Godoy F. Hargreaves K. M. (2007). Regenerative endodontics: a review of current status and a call for action. J. Endod. 33, 377–390. 10.1016/j.joen.2006.09.013 17368324

[B97] Nakagawa M. Koyanagi M. Tanabe K. Takahashi K. Ichisaka T. Aoi T.. (2008). Generation of induced pluripotent stem cells without Myc from mouse and human fibroblasts. Nat. Biotechnol. 26, 101–106. 10.1038/nbt1374 18059259

[B98] Ning F. Guo Y. Tang J. Zhou J. Zhang H. Lu W.. (2010). Differentiation of mouse embryonic stem cells into dental epithelial-like cells induced by ameloblasts serum-free conditioned medium. Biochem. Biophys. Res. Commun. 394, 342–347. 10.1016/j.bbrc.2010.03.007 20206604

[B99] Oda Y. Yoshimura Y. Ohnishi H. Tadokoro M. Katsube Y. Sasao M.. (2010). Induction of pluripotent stem cells from human third molar mesenchymal stromal cells. J. Biol. Chem. 285, 29270–29278. 10.1074/jbc.M109.055889 20595386 PMC2937959

[B100] Okay D. Al Shetawi A. H. Moubayed S. P. Mourad M. Buchbinder D. Urken M. L. (2016). Worldwide 10-year systematic review of treatment trends in fibula free flap for mandibular reconstruction. J. Oral Maxillofac. Surg. 74, 2526–2531. 10.1016/j.joms.2016.06.170 27400143

[B101] Okita K. Nakagawa M. Hyenjong H. Ichisaka T. Yamanaka S. (2008). Generation of mouse induced pluripotent stem cells without viral vectors. Science 322, 949–953. 10.1126/science.1164270 18845712

[B102] Okuda A. Horii-Hayashi N. Sasagawa T. Shimizu T. Shigematsu H. Iwata E.. (2017). Bone marrow stromal cell sheets may promote axonal regeneration and functional recovery with suppression of glial scar formation after spinal cord transection injury in rats. J. Neurosurg. Spine 26, 388–395. 10.3171/2016.8.SPINE16250 27885959

[B103] Otsu K. Kishigami R. Oikawa-Sasaki A. Fukumoto S. Yamada A. Fujiwara N.. (2012). Differentiation of induced pluripotent stem cells into dental mesenchymal cells. Stem Cells Dev. 21, 1156–1164. 10.1089/scd.2011.0210 22085204

[B104] Otsu K. Kumakami-Sakano M. Fujiwara N. Kikuchi K. Keller L. Lesot H.. (2014). Stem cell sources for tooth regeneration: current status and future prospects. Front. Physiol. 5:36. 10.3389/fphys.2014.00036 24550845 PMC3912331

[B105] Ozcan M. Schoonbeek G. Gokce B. Comlekoglu E. Dundar M. (2010). Bond strength comparison of amalgam repair protocols using resin composite in situations with and without dentin exposure. Oper. Dent. 35, 655–662. 10.2341/10-091-L 21180005

[B106] Paino F. La Noce M. Tirino V. Naddeo P. Desiderio V. Pirozzi G.. (2014). Histone deacetylase inhibition with valproic acid downregulates osteocalcin gene expression in human dental pulp stem cells and osteoblasts: evidence for HDAC2 involvement. Stem Cells 32, 279–289. 10.1002/stem.1544 24105979 PMC3963447

[B107] Panduwawala C. P. Zhan X. Dissanayaka W. L. Samaranayake L. P. Jin L. Zhang C. (2016). *In vivo* periodontal tissue regeneration by periodontal ligament stem cells and endothelial cells in three-dimensional cell sheet constructs. J. Periodont. Res. [Epub ahead of print]. 10.1111/jre.12405 27495271

[B108] Phipps M. C. Xu Y. Bellis S. L. (2012). Delivery of platelet-derived growth factor as a chemotactic factor for mesenchymal stem cells by bone-mimetic electrospun scaffolds. PLoS ONE 7:e40831. 10.1371/journal.pone.0040831 22808271 PMC3395644

[B109] Potdar P. D. Jethmalani Y. D. (2015). Human dental pulp stem cells: applications in future regenerative medicine. World J. Stem Cells 7, 839–851. 10.4252/wjsc.v7.i5.839 26131314 PMC4478630

[B110] Rada C. Jarvis J. M. Milstein C. (2002). AID-GFP chimeric protein increases hypermutation of Ig genes with no evidence of nuclear localization. Proc. Natl. Acad. Sci. U.S.A. 99, 7003–7008. 10.1073/pnas.092160999 12011459 PMC124518

[B111] Rajan A. Eubanks E. Edwards S. Aronovich S. Travan S. Rudek I.. (2014). Optimized cell survival and seeding efficiency for craniofacial tissue engineering using clinical stem cell therapy. Stem Cells Transl. Med. 3, 1495–1503. 10.5966/sctm.2014-0039 25378653 PMC4250207

[B112] Rosa V. Zhang Z. Grande R. H. Nor J. E. (2013). Dental pulp tissue engineering in full-length human root canals. J. Dent. Res. 92, 970–975. 10.1177/0022034513505772 24056227 PMC3797540

[B113] Schipani E. Maes C. Carmeliet G. Semenza G. L. (2009). Regulation of osteogenesis-angiogenesis coupling by HIFs and VEGF. J. Bone Miner. Res. 24, 1347–1353. 10.1359/jbmr.090602 19558314 PMC3276346

[B114] Seo B. M. Miura M. Gronthos S. Bartold P. M. Batouli S. Brahim J.. (2004). Investigation of multipotent postnatal stem cells from human periodontal ligament. Lancet 364, 149–155. 10.1016/S0140-6736(04)16627-0 15246727

[B115] Sequeira-Byron P. Fedorowicz Z. Carter B. Nasser M. Alrowaili E. F. (2015). Single crowns versus conventional fillings for the restoration of root-filled teeth. Cochrane Database Syst Rev. 16:CD009109. 10.1002/14651858.cd009109.pub3 26403154 PMC7111426

[B116] Shafiee A. Atala A. (2016). Printing technologies for medical applications. Trends Mol. Med. 22, 254–265. 10.1016/j.molmed.2016.01.003 26856235

[B117] Smith B. T. Shum J. Wong M. Mikos A. G. Young S. (2015). Bone tissue engineering challenges in oral and maxillofacial surgery. Adv. Exp. Med. Biol. 881, 57–78. 10.1007/978-3-319-22345-2_4 26545744

[B118] Soares T. R. Fidalgo T. K. Quirino A. S. Ferreira D. M. Chianca T. K. Risso P. A.. (2016). Is caries a risk factor for dental trauma? A systematic review and meta-analysis. Dent. Traumatol. 33, 4–12. 10.1111/edt.12295 27439566

[B119] Sun J. B. Wang J. G. Pefanis E. Chao J. M. Rothschild G. Tachibana I.. (2015). Transcriptomics Identify CD9 as a marker of murine IL-10-competent regulatory B cells. Cell Rep. 13, 1110–1117. 10.1016/j.celrep.2015.09.070 26527007 PMC4644501

[B120] Sun J. Li Y. Liang X. L. Wang P. C. (2011). Bacterial magnetosome: a novel biogenetic magnetic targeted drug carrier with potential multifunctions. J. Nanomaterials. 2011, 469031–469043. 10.1155/2011/469031 22448162 PMC3310401

[B121] Sung I. Y. Son H. N. Ullah I. Bharti D. Park J. M. Cho Y. C.. (2016). Cardiomyogenic differentiation of human dental follicle-derived stem cells by suberoylanilide hydroxamic acid and their *in vivo* homing property. Int. J. Med. Sci. 13, 841–852. 10.7150/ijms.16573 27877076 PMC5118755

[B122] Szabo E. Rampalli S. Risueno R. M. Schnerch A. Mitchell R. Fiebig-Comyn A.. (2010). Direct conversion of human fibroblasts to multilineage blood progenitors. Nature 468, 521–526. 10.1038/nature09591 21057492

[B123] Takahashi K. Yamanaka S. (2006). Induction of pluripotent stem cells from mouse embryonic and adult fibroblast cultures by defined factors. Cell 126, 663–676. 10.1016/j.cell.2006.07.024 16904174

[B124] Tamaoki N. Takahashi K. Tanaka T. Ichisaka T. Aoki H. Takeda-Kawaguchi T.. (2010). Dental pulp cells for induced pluripotent stem cell banking. J. Dent. Res. 89, 773–778. 10.1177/0022034510366846 20554890

[B125] Tantbirojn D. Pfeifer C. S. Braga R. R. Versluis A. (2011). Do low-shrink composites reduce polymerization shrinkage effects?” J. Dent. Res. 90, 596–601. 10.1177/0022034510396217 21282725

[B126] Tatullo M. Marrelli M. Paduano F. (2015). The regenerative medicine in oral and maxillofacial surgery: the most important innovations in the clinical application of mesenchymal stem cells. Int. J. Med. Sci. 12, 72–77. 10.7150/ijms.10706 25552921 PMC4278878

[B127] Theocharidou A. Bakopoulou A. Kontonasaki E. Papachristou E. Hadjichristou C. Bousnaki M.. (2016). Odontogenic differentiation and biomineralization potential of dental pulp stem cells inside Mg-based bioceramic scaffolds under low-level laser treatment. Lasers Med. Sci. 32, 201–210. 10.1007/s10103-016-2102-9 27785631

[B128] Trowbridge H. O. (1981). Pathogenesis of pulpitis resulting from dental caries. J. Endod. 7, 52–60. 10.1016/S0099-2399(81)80242-7 6938624

[B129] Ueda M. Yamada Y. Ozawa R. Okazaki Y. (2005). Clinical case reports of injectable tissue-engineered bone for alveolar augmentation with simultaneous implant placement. Int. J. Periodontics Restorative Dent. 25, 129–137. 10.1016/j.prosdent.2005.05.019 15839589

[B130] Uskokovic V. (2015). Amelogenin in enamel tissue engineering. Adv. Exp. Med. Biol. 881, 237–254. 10.1007/978-3-319-22345-2_13 26545753 PMC4773195

[B131] Vierbuchen T. Ostermeier A. Pang Z. P. Kokubu Y. Sudhof T. C. Wernig M. (2010). Direct conversion of fibroblasts to functional neurons by defined factors. Nature 463, 1035–1041. 10.1038/nature08797 20107439 PMC2829121

[B132] Wada N. Wang B. Lin N. H. Laslett A. L. Gronthos S. Bartold P. M. (2011). Induced pluripotent stem cell lines derived from human gingival fibroblasts and periodontal ligament fibroblasts. J. Periodont. Res. 46, 438–447. 10.1111/j.1600-0765.2011.01358.x 21443752

[B133] Wang Y. Zhou L. Li C. Xie H. Lu Y. Wu Y.. (2015). Bone marrow-derived cells homing for self-repair of periodontal tissues: a histological characterization and expression analysis. Int. J. Clin. Exp. Pathol. 8, 12379–12389. 26722424 PMC4680369

[B134] Weissman I. L. (2000). Stem cells: units of development, units of regeneration, and units in evolution. Cell 100, 157–168. 10.1016/S0092-8674(00)81692-X 10647940

[B135] Wen Y. Wang F. Zhang W. Li Y. Yu M. Nan X.. (2012). Application of induced pluripotent stem cells in generation of a tissue-engineered tooth-like structure. Tissue Eng. Part A 18, 1677–1685. 10.1089/ten.tea.2011.0220 22676377 PMC3419858

[B136] Wijbenga J. G. Schepers R. H. Werker P. M. Witjes M. J. Dijkstra P. U. (2016). A systematic review of functional outcome and quality of life following reconstruction of maxillofacial defects using vascularized free fibula flaps and dental rehabilitation reveals poor data quality. J. Plast. Reconstr. Aesthet. Surg. 69, 1024–1036. 10.1016/j.bjps.2016.05.003 27292287

[B137] Windisch P. Stavropoulos A. Molnar B. Szendroi-Kiss D. Szilagyi E. Rosta P.. (2012). A phase IIa randomized controlled pilot study evaluating the safety and clinical outcomes following the use of rhGDF-5/beta-TCP in regenerative periodontal therapy. Clin. Oral Investig. 16, 1181–1189. 10.1007/s00784-011-0610-3 21887500

[B138] Wu T. Liu Y. Fan Z. Xu J. Jin L. Gao Z.. (2015). miR-21 Modulates the immunoregulatory function of bone marrow mesenchymal stem cells through the PTEN/Akt/TGF-β1 pathway. Stem Cells 33, 3281–3290. 10.1002/stem.2081 26086742

[B139] Xu A. Liu Y. Chen W. Wang J. Xue Y. Huang F.. (2016). TGF-beta-Induced regulatory T cells directly suppress B cell responses through a noncytotoxic mechanism. J. Immunol. 196, 3631–3641. 10.4049/jimmunol.1501740 27001954 PMC4868785

[B140] Xu J. Wang D. Liu D. Fan Z. Zhang H. Liu O.. (2012). Allogeneic mesenchymal stem cell treatment alleviates experimental and clinical Sjogren syndrome. Blood 120, 3142–3151. 10.1182/blood-2011-11-391144 22927248 PMC3471521

[B141] Yadlapati M. Biguetti C. Cavalla F. Nieves F. Bessey C. Bohluli P.. (2017). Characterization of a vascular endothelial growth factor–loaded bioresorbable delivery system for pulp regeneration. J. Endod. 43, 77–83. 10.1016/j.joen.2016.09.022 27939739

[B142] Yan X. Qin H. Qu C. Tuan R. S. Shi S. Huang G. T. (2010). iPS cells reprogrammed from human mesenchymal-like stem/progenitor cells of dental tissue origin. Stem Cells Dev. 19, 469–480. 10.1089/scd.2009.0314 19795982 PMC2851830

[B143] Yoshida K. Sato J. Takai R. Uehara O. Kurashige Y. Nishimura M.. (2015). Differentiation of mouse iPS cells into ameloblast-like cells in cultures using medium conditioned by epithelial cell rests of Malassez and gelatin-coated dishes. Med. Mol. Morphol. 48, 138–145. 10.1007/s00795-014-0088-6 25319805

[B144] Yu J. Hu K. Smuga-Otto K. Tian S. Stewart R. Slukvin I. I.. (2009). Human induced pluripotent stem cells free of vector and transgene sequences. Science 324, 797–801. 10.1126/science.1172482 19325077 PMC2758053

[B145] Yu J. Vodyanik M. A. Smuga-Otto K. Antosiewicz-Bourget J. Frane J. L. Tian S.. (2007). Induced pluripotent stem cell lines derived from human somatic cells. Science 318, 1917–1920. 10.1126/science.1151526 18029452

[B146] Yusa K. Yamamoto O. Iino M. Takano H. Fukuda M. Qiao Z.. (2016b). Eluted zinc ions stimulate osteoblast differentiation and mineralization in human dental pulp stem cells for bone tissue engineering. Arch. Oral Biol. 71, 162–169. 10.1016/j.archoralbio.2016.07.010 27521529

[B147] Yusa K. Yamamoto O. Takano H. Fukuda M. Iino M. (2016a). Zinc-modified titanium surface enhances osteoblast differentiation of dental pulp stem cells *in vitro*. Sci. Rep. 6:29462. 10.1038/srep29462 27387130 PMC4937451

[B148] Zhang Q. Shi S. Liu Y. Uyanne J. Shi Y. Shi S.. (2009). Mesenchymal stem cells derived from human gingiva are capable of immunomodulatory functions and ameliorate inflammation-related tissue destruction in experimental colitis. J. Immunol. 183, 7787–7798. 10.4049/jimmunol.0902318 19923445 PMC2881945

[B149] Zhang X. H. Zhang C. G. Lin Y. H. Hu P. H. Shen Y. Wang K.. (2016). Nanocomposite membranes enhance bone regeneration through restoring physiological electric microenvironment. ACS Nano 10, 7279–7286. 10.1021/acsnano.6b02247 27389708

[B150] Zhou H. Wu S. Joo J. Y. Zhu S. Han D. W. Lin T.. (2009). Generation of induced pluripotent stem cells using recombinant proteins. Cell Stem Cell 4, 381–384. 10.1016/j.stem.2009.04.005 19398399 PMC10182564

[B151] Zhou Q. Brown J. Kanarek A. Rajagopal J. Melton D. A. (2008). *in vivo* reprogramming of adult pancreatic exocrine cells to beta-cells. Nature 455, 627–632. 10.1038/nature07314 18754011 PMC9011918

